# SWATH-MS based quantitative proteomics analysis reveals that curcumin alters the metabolic enzyme profile of CML cells by affecting the activity of miR-22/IPO7/HIF-1α axis

**DOI:** 10.1186/s13046-018-0843-y

**Published:** 2018-07-25

**Authors:** Francesca Monteleone, Simona Taverna, Riccardo Alessandro, Simona Fontana

**Affiliations:** 1 Department of Biopathology and Medical Biotechnologies - Section of Biology and Genetics, University of Palermo, Palermo, Italy; 2 Institute of Biomedicine and Molecular Immunology (IBIM), National Research Council, Palermo, Italy

**Keywords:** Curcumin, CML cells, SWATH-MS, miR-22/IPO7/HIF-1α axis

## Abstract

**Background:**

Chronic myelogenous leukemia (CML) is a myeloproliferative disorder caused by expression of the chimeric BCR-ABL tyrosine kinase oncogene, resulting from the t(9;22) chromosomal translocation. Imatinib (gleevec, STI-571) is a selective inhibitor of BCR-ABL activity highly effective in the treatment of CML. However, even though almost all CML patients respond to treatment with imatinib or third generation inhibitors, these drugs are not curative and need to be taken indefinitely or until patients become resistant. Therefore, to get a definitive eradication of leukemic cells, it is necessary to find novel therapeutic combinations, for achieving greater efficacy and fewer side effects.

Curcumin is an Indian spice with several therapeutic properties: anti-oxidant, analgesic, anti-inflammatory, antiseptic and anti-cancer. In cancer disease, it acts by blocking cell transformation, proliferation, and invasion and by inducing cell apoptosis.

**Methods:**

In the present study, the effect of a sub-toxic dose of curcumin on K562 cells was evaluated by using the technique of Sequential Window Activation of All Theoretical Mass Spectra (SWATH-MS). Bioinformatic analysis of proteomic data was performed to highlight the pathways mostly affected by the treatment. The involvement of Hypoxia inducible factor 1 α (HIF-1α) was assayed by evaluating its activation status and the modulation of importin 7 (IPO7) and miR-22 was assessed by quantitative PCR and western blot analysis. Finally, K562 cells transfected with miR-22 inhibitor were used to confirm the ability of curcumin to elicit miR-22 expression.

**Results:**

Our findings revealed that the most relevant effect induced by curcumin was a consistent decrease of several proteins involved in glucose metabolism, most of which were HIF-1α targets, concomitant with the up-regulation of functional and structural mitochondrial proteins. The mechanism by which curcumin affects metabolic enzyme profile was associated with the reduction of HIF-1α activity, due to the miR-22-mediated down-regulation of IPO7 expression. Finally, the ability of curcumin to enhance in vitro the efficiency of imatinib was reported.

**Conclusions:**

In summary, our data indicates that the miR-22/IPO7/HIF-1α axis may be considered as a novel molecular target of curcumin adding new insights to better define therapeutic activity and anticancer properties of this natural compound. The MS proteomic data have been deposited to the ProteomeXchange with identifier <PXD007771>.

**Electronic supplementary material:**

The online version of this article (10.1186/s13046-018-0843-y) contains supplementary material, which is available to authorized users.

## Background

Chronic myelogenous leukemia (CML) is a myeloproliferative neoplasm marked by the presence of shortened Philadelphia chromosome (Ph) that results from a reciprocal translocation between chromosome 9 and chromosome 22 [(9;22) (q34;q11)]. The molecular consequence of this translocation is the generation of BCR-ABL fusion oncogene. The protein encoded by this fusion gene is the constitutively active tyrosine kinase BCR-ABL, which drives the malignant process in CML cells and represents the primary target of therapy [1].

Although the tyrosine kinase inhibitors (TKI) as imatinib mesylate (imatinib, Gleevec) or third generation inhibitors, are effective in controlling CML and preventing progression to blast crisis, they are not curative. Indeed, BCR-ABL transcript can be detected in patients who achieve complete cytogenetic responses [2], and the cessation of TKI therapy may result in disease relapse also in patients with undetectable level of BCR-ABL transcript [3]. These effects are due to the incapability of TKIs to eliminate leukemia stem cells (LSCs) whose survival is probably supported by kinase-independent pathways [4]. Several experimental evidences highlighted that the upregulation of hypoxia-inducible factor 1 α (HIF-1α) supports LSCs maintenance and potency [4–7], thus indicating that the identification of new drugs targeting HIF-1α may be crucial for developing combined therapeutic strategies to effectively treat patients with CML.

A high number of preclinical studies have shown the relevant role that natural compounds can have in cancer prevention as well as, if used in tandem with conventional therapy, in cancer treatment [8]. Among these natural agents, curcumin, a natural polyphenol compound extracted from the rhizome of *Curcuma longa*, is reported to inhibit tumor growth through regulating multiple signaling pathways involved in cell proliferation, survival, apoptosis, inflammation and angiogenesis [9].

In our previous papers we demonstrated that curcumin treatment of CML cells caused a selective sorting of active miR-21 in exosomes and a concomitant decrease of this miRNA in the cells thus leading to the upregulation of PTEN that in turn caused a decrease of AKT phosphorylation and VEGF expression and release. Furthermore, we showed that addition of curcumin to CML cells caused a downregulation of BCR-ABL expression through the cellular increase of miR-196b. Moreover, we observed that animals treated with curcumin, developed smaller tumors compared to control mice and that exosomes isolated from their plasma were enriched in miR-21 [10, 11].

In this study, we employed the proteomic quantitative analytic method SWATH (Sequential Window Acquisition of all THeoretical fragment-ion spectra) [12] to examine how curcumin affected the protein profile of K562 cells. Our findings revealed that curcumin treatment, in CML cells, induced an increase of several mitochondrial structural and functional proteins and a simultaneous consistent decrease of several proteins involved in glucose metabolism, most of which were HIF-1α targets. According to this observation, we found that curcumin was able induce a significant reduction of HIF-1α activity in comparison to untreated K562 cells. Moreover, the bioinformatics analysis of protein-expression data allowed us to identify miR-22 and its target Importin 7 (IPO7) as possible molecular mediators of the effects observed in curcumin-treated CML cells. Our findings reveal the ability of curcumin to up-regulate the expression levels of miR-22 and down-regulate the IPO7 expression, thus affecting the cytoplasm-to-nucleus shuttling of HIF-1α. We also demonstrated that the curcumin-induced up-regulation of miR22 may increase the intrinsic imatinib sensitivity of K562 cells, providing new insights for reducing excessive side effects in patients using the current fixed dosing strategy.

## Methods

### Cell culture, reagents and treatments

The human CML cell lines K562 and LAMA84 (DSMZ, Braunschweig, Germany) were cultured in RPMI 1640 medium (Euroclone, UK), supplemented with 10% fetal bovine serum, 2 mM L-glutamine, 100 U/ml penicillin, and 100 μg/ml streptomycin (Euroclone, UK).

Imatinib (Selleck) was prepared as 5 mM stock solution in sterile phosphate-buffered saline (PBS); curcumin was prepared as 100 mM stock solution in 0.1% DMSO; working solutions of both curcumin and imatinib were performed in culture medium the same day of use. For the following experiments cells were treated with 20 μM curcumin (Curcu-K562) and/or escalating doses of imatinib (Im-K562) for 24 h. In the assays with curcumin, control cells (Ctrl-K562) were treated with DMSO percentage used in working solution (Ctrl-K562).

All other reagents were purchased from Sigma (St. Louis, MO), if not otherwise cited.

### Proteomic analyses: Sample preparation, SWATH-MS and data analysis

#### Protein extraction and digestion

Chemicals used for protein extraction and digestion were of analytical grade, and Milli-Q water was employed in all buffers and solutions. K562 treated with only DMSO or with 20 μM of curcumin (respectively Ctrl-K562 and Curcu-K562) were lysed in RIPA buffer (NaCl 5 M, Tris HCl pH 7.6 1 M, 5% Triton X-100) containing protease and phosphatase inhibitors for 90 min in ice; lysates were centrifuged at 14000 g for 12 min at 4 °C and protein concentration was determined by Bradford method. Before performing tryptic digestion, 250 μg of each cell lysate were precipitated using the 2-D Clean-Up Kit (Amersham) to remove Triton and other contaminants used for cell lysis; purified proteins were suspended buffer containing Tris HCl (100 mM) and urea (8 M) and subjected to in-solution digestion. Reduction was performed with 2 mM DTT for 30 min at 37 °C and alkylation with 8 mM IAA for 30 min in the dark at 37 °C. Prior to trypsin addition, sample was diluted with five-volumes of 100 mM NH_4_HCO_3_ pH 8.5. Proteins were digested using 4 μg of sequencing-grade modified porcine trypsin (Pierce) (1:60, *w*/w, trypsin to protein ratio), in presence of 2 mM CaCl_2_. After overnight incubation, digestion was stopped by adding 50 μl of 2.5% trifluoroacetic acid (TFA). Once concentrated with a speed vacuum centrifuge, before injection, extracted peptides were desalted by solid phase extraction using C18 Macrospin Columns. C18 columns were conditioned with acetonitrile (ACN) and rinsed with water; peptides were eluted with 70% ACN/H_2_O (70:30, *v*/v) containing 0.1% formic acid (FA), and were dried, to be then re-suspended in 5% ACN/H_2_O (5:95, v/v) containing 0.1% FA. For each sample, two biological replicates were prepared and used for the following proteomic analysis.

#### Generation of the reference spectral library

Approximately 2 μg of pooled tryptic peptides from each biological replicate of Ctrl-K562 and Curcu-K562 were subjected to Data Dependent Acquisition (DDA) analysis. The resulting list of protein/peptides was used for construction of the SWATH reference spectral library. The sample was analyzed via reverse-phase high-pressure liquid chromatography electrospray ionization tandem mass spectrometry (RP-HPLC-ESI-MS/MS) using a TripleTOF® 5600 mass spectrometer (AB SCIEX; Framingham, US). The mass spectrometer was coupled to a nanoLC Eksigent 425 system (AB SCIEX; Framingham, US). RP-HPLC was performed with a trap and elution configuration using a Nano cHiPLC Trap column 200 μm × 0.5 mm ChromXP C18-CL 3 μm 120 Å and a Nano cHiPLC column 75 μm × 15 cm ChromXP C18-CL 3 μm 120 Å. The reverse-phase LC solvents were: solvent A (0.1% formic acid in water) and solvent B (2% water and 0.1% formic acid in acetonitrile). The sample was loaded in the trap column at a flow rate of 5 μl/min for 10 min in a solvent containing 2% acetonitrile and 0.1% *v*/v TFA in water and eluted at a flow rate of 300 nl/min using the following gradient method: 1) solvent B from 10 to 28% within 120 min and to 60% within 30 min; 2) phase B to 95.2% within 2 min; 3) phase B decreased to 94.8% for 10 min (to rinse the column); 4) phase B decreased to 10% over 2 min. Finally, the column was equilibrated for 36 min (200 min total run time). The eluting peptides were on-line sprayed in the Triple TOF 5600 Plus mass spectrometer, that it is controlled by Analysts 1.6.1 software (AB SCIEX, Toronto, Canada).

For DDA run, the mass range for MS scan was set to *m*/*z* 350–1250 and the MS/MS scan mass range was set to *m*/*z* 230–1500. Using the mass spectrometer, a 0.25 s survey scan (MS) was performed, and the top 25 ions were selected for subsequent MS/MS experiments employing an accumulation time of 0.15 s per MS/MS experiment for a total cycle time of 4.0504 s. Precursor ions were selected in high resolution mode (> 30,000), tandem mass spectra were recorded in high sensitivity mode (resolution > 15,000). The selection criteria for parent ions included an intensity of greater than 50 cps and a charge state ranging from + 2 to + 5. A 15 s dynamic exclusion was used. The ions were fragmented in the collision cell using rolling collision energy, and CES was set to 2.

The DDA MS raw file was subjected to database searches using ProteinPilot™ 4.5 software (AB SCIEX; Framingham, US) with the Paragon algorithm by using the following parameters: iodoacetamide cysteine alkylation, digestion by trypsin and no special factors. The search was conducted through identification efforts in a UniProt database (downloaded in July 2014, with 137,216 protein sequence entries) containing whole *Homo sapiens* proteins. A false discovery rate analysis was performed.

#### SWATH-MS analysis and targeted data extraction

The two biological replicates of Ctrl-K562 and Curcu-K562 (2 μg each) were twice run and subjected to the cyclic data independent acquisition (DIA) of mass spectra. Data were acquired by repeatedly cycling through 34 consecutive 25-Da precursor isolation windows (swaths). For these experiments, the mass spectrometer was operated using a 0.05 s survey scan (MS). The subsequent MS/MS experiments were performed across the mass range of 350 to 1250 m/z on all precursors in a cyclic manner using an accumulation time of 0.0898 s per SWATH window for a total cycle time of 3.3335 s. Ions were fragmented for each MS/MS experiment in the collision cell using rolling collision energy, and CES was set to 15. Spectral alignment and targeted data extraction of DIA data files were performed with PeakView v.2.2 SWATH Processing MicroApp v2.0 (AB SCIEX; Framingham, US) by using the reference spectral library generated as above described. All eight DIA files were loaded in one comparison group in unison and processed as reported by Li H. et al. [13] with the following modifications: up to ten peptides/protein and up to seven transitions/peptide. The area under the intensity curve for individual ions of a targeted peptide were summed to represent the peptide and the areas of the corresponding peptides were summed to represent the targeted proteins. These areas were used for relative quantification and statistics analysis. For each protein, seven individual ion intensities were summed for obtaining peptide intensity, ten peptides intensities were summed for obtaining protein intensity. The mass spectrometry proteomics data have been deposited to the ProteomeXchange Consortium (http://proteomecentral.proteomexchange.org) [14] via the PRIDE partner repository [15] with the dataset identifier <PXD007771>.

### Statistical analysis, gene ontology analysis, functional networks and pathway mapping

The protein list with FDR lower than 5% generated by analyzing SWATH data with PeakView 2.2, was exported to MarkerView 1.2.1 (AB SCIEX; Framingham, US) for normalization of protein intensity (peak area) using the total area sums algorithm and *t*-test analysis [13, 16]. Mean of all biological and technical replicates was used to compare proteins of Ctrl-K562 and Curcu-K562. Fold Change (FC) Curcu-K562 vs Ctrl-K562 thresholds at ±1.5 with a corrected *p*-value inferior to 0.05 were used to consider a protein up or down-regulated. GraphPad Prism 7.00 for Windows was used for (i) performing the *p*-value Benjamini-Yekutieli (BY-Pvalue) correction (13); (ii) to make a volcano plot scaling in which the FC was transformed using the log2 function, so that the data is centered on zero, while the BY corrected *p*-value was −log10 transformed. The Graphical Proteomics Data Explorer (GProX), a freely available complete software platform (14) was used to calculate the linear regression of repeats. The analysis of coefficients of variation for replicates was performed using Microsoft Excel 2010. The expression-based heat map was obtained by using the Heatmapper freely available web server (http://www.heatmapper.ca). The Gene Ontology (GO) and KEGG (Kyoto Encyclopedia of Genes and Genomes) analysis of proteins up- and down-regulated following treatment with curcumin (respectively Curcu-UpRegProteins and Curcu-DownRegProteins) were initially performed using the stand-alone enrichment analysis tool FunRich (Functional Enrichment analysis tool; http://www.funrich.org) [17]. The ClueGO v2.3.3 + CluePedia v1.3.3, a Cytoscape v3.4.0 plug-in was used to visualize the non-redundant GO terms and pathways in functionally organized networks reflecting the relations between the biological terms based on the similarity of their linked gene/proteins [18]. In order to make cluster/group comparison and highlight functional differences, the two protein groups mentioned above were uploaded in ClueGO as two separate clusters using the Cytoscape environment [19].

For the enrichment of biological terms and groups, we used the two-sided (Enrichment/Depletion) tests based on the hyper-geometric distribution. We set the statistical significance to 0.05 (*p* ≤ 0.05), and we used the Benjamini-Hochberg adjustment to correct the *p*-value for the terms and the groups created by ClueGO. We used fusion criteria to diminish the redundancy of the terms shared by similar associated proteins, which allows one to maintain the most representative parent or child term and the used parameters were: kappa score threshold set to 0.4; GO tree interval: 3–8; GO Term Fusion; Leading Group: Highest Significance; % of Group Merge: 50.

### Quantitative polymerase chain reaction (qPCR) for miR-22

The expression of miR-22 was tested by miScript PCR System (QIAGEN, Hilden, Germany). The RNAspin Mini (GE Healthcare Science, Uppsala, Sweden) was used to isolate total RNA from K562 and LAMA84 cells treated or not with curcumin. Reverse transcription reactions were performed using miScript II RT Kit (QIAGEN, Hilden, Germany) as described by the manufacturer’s instructions. We used miScript HiSpec Buffer for cDNA synthesis to detect mature miRNA. Quantitative Real Time PCR was performed using miScript SYBR Green PCR Kit (QIAGEN, Hilden, Germany). Mature miR-22-3p (mature miRNA sequence 5’-AAGCUGCCAGUUGAAGAACUGU-3′) was detected by miScript Primer Assay (MIMAT0000077; QIAGEN, Hilden, Germany) according to manufacturer’s instructions. RNU6–2 was used as endogenous control. Expression levels of miRNAs were determined using the comparative Ct method to calculate changes in Ct and ultimately fold and percent change. An average Ct value for each RNA was obtained from triplicate reactions.

### qPCR for HIF-1α and IPO7

Total cellular RNA was isolated from K562 and LAMA84 cells treated with curcumin using the RNAspin Mini (GE Healthcare Science, Uppsala, Sweden). For HIF-1α and Importin 7 (IPO7) mRNA detection, 1 μg of total RNA was reverse transcribed using the High Capacity cDNA Archive kit (Life Technologies, Carlsbad, California, U.S.), according to manufacturer’s instructions. HIF-1α and IPO7 transcript levels were measured by quantitative SYBER®Green real time PCR; reactions were carried out in a total volume of 20 μl containing 2× SYBR®Green I Master Mix (Applied Biosystems), 2 μl cDNA and 300 nM forward and reverse primers. Primers sequence were: GAPDH (5′ATGGGGAAGGTGAAGGTCG3′; 5′GGGTCATTGATGGCAACAATAT3′), HIF-1α (5’ TGATTGCATCTCCATCTCCTACC3’; 5’GACTCAAAGCGACAGATAACACG3’) and IPO7 (5’TGGGACCTGATCATGCAACC3’; 5’AGCTGCCTTCATGACATCCC3’). All reagents were from Invitrogen (Foster City, CA, USA). Real-time PCR was performed in duplicates for each data point. Relative changes in gene expression between control and treated samples were determined with the ΔΔCt method. Changes in the target mRNA content relative to GAPDH were determined using the comparative Ct method as described in the previous paragraph.

### Transfection of K562 cells with miR-22 inhibitor

Transfection of miScript miR-22-3p inhibitor (MIN0000077, QIAGEN, Hilden, Germany) in K562 cells (QIAGEN, Hilden, Germany) was performed according Fast-Forward Transfection protocol (QIAGEN, Hilden, Germany). 6 × 10^4^ K562 cells per well were seeded in a 24-well plate in 500 μl of RPMI. miScript miR-22 (2’-O-Me-miR-22) (2 μM) were diluted in 100 μl culture medium without serum to obtain a final 5 nM miRNA concentration. The cells were transfected using HiPerFect Transfection Reagent (QIAGEN, Hilden, Germany) according to manufacturer’s instructions for 18 h. MiScript Inhibitor Negative Control (QIAGEN, Hilden, Germany) was used as negative control as indicated by manufacture’s technical specifications. Transfection efficiency was evaluated by quantitative Real Time PCR.

### HIF1*α* transcription activation assay

K562 and LAMA84 cells treated with 20 μM of curcumin were harvested after 24 h of incubation. K562 cells were also transfected or not with miR-22 inhibitor. Nuclear extracts were prepared by using the Nuclear Extract Kit from Activemotif (#40010 - Activemotif, Carlsbad, CA, USA) according to the manufacturer’s instructions. The transcriptional activity of HIF-1α was assayed by an ELISA-based kit (#47096 - TransAM Kit, Activemotif, Carlsbad, CA, USA) according to the manufacturer’s instructions. Nuclear extract samples were added to the coated plate and analyzed at 450 nm with Gen5 Microplate Collection & Analysis Software Data (BioTek Instruments, Inc.®). HIF-1α activity was expressed as Absorbance value.

### Western blot

Total cell lysates or nuclear/cytoplasm fractions were resolved in 6 or 8% SDS-PAGE and analyzed by Western Blot. Primary antibodies used in the experiments were: anti-IPO7 (1:500; ThermoFischer), anti-HIF-1α (1:1000; Millipore) and anti-actin beta (1:500; Cell Signaling Technology, Beverly, MA). Secondary antibodies (anti-mouse or anti-rabbit depending on primary antibody) were Alexa Fluor or HRP conjugated. When HRP conjugated secondary antibodies were used, protein bands were detected using Amersham ECL Western Blotting Detection Reagent; protein bands were finally visualized using Chemidoc (BioRad). The signal intensity of each band was calculated with Image J software (http://rsbweb.nih.gov/ij/).

### Proliferation assay (MTT assay)

Methyl-thiazol-tetrazolium (MTT) assay was done as previously described [20, 21]. K562 and LAMA84 cells were seeded in 96-well plates (2–5 × 103 cells/well) and incubated for 24 h with 10, 20, 40 μM curcumin for 24 h.

For co-treatment experiments, K562 cells were seeded in 96-well plates (2–5 × 103 cells/well) and incubated for 24 h with 20 μM curcumin (Curcu-K562) and/or escalating doses of imatinib (Im-K562). In order to assess the synergism between curcumin and imatinib on viability, both K562 and LAMA84 cells were treated for 48 h with curcumin alone (5, 10, 20, 40, 50 μM), imatinib alone (0.1, 0.2, 0.5, 1 and 5 μM) or imatinib + curcumin combination (imatinib at concentrations of 0.1, 0.2, 0.5, 1 and 5 μM; 20 μM curcumin held constant). Cell proliferation was assessed using a MTT assay. Control cells were seeded in absence of curcumin and with appropriate percentage of DMSO. Means and standard deviations were generated from three independent experiments. Graphs and statistical analysis were performed by using Microsoft Excel 2010. For synergism determination, percent cell survival results were converted to Fraction Affected (FrAf) as follows: FrAf = 0 represents 100% viability; FrAf = 1 represents 0% viability. Imtinib + curcumin interaction was assessed by Combination Index (CI) analysis using the CompuSyn software program (ComboSyn, Inc., Paramus, NJ) based on the Chou-Talalay equation [22, 23]. CI index of less than 1 was considered to indicate a synergistic interaction.

## Results

**Fig. 1:**
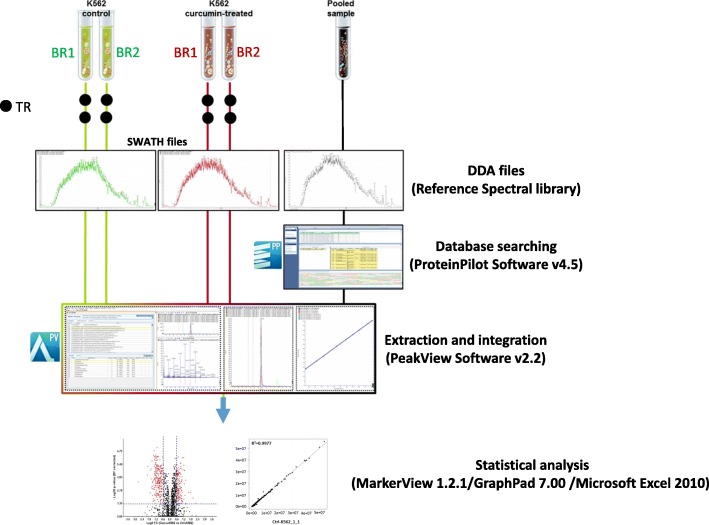
Experimental design and workflow for quantitative proteomics analysis of Ctrl-K562 and Curcu-K562 cells. The SWATH-MS analysis was performed using a Triple TOF 5600+ system equipped with a Nanospray III interface and coupled to an Eksigent nanoLC 425. For quantitative analysis, 1 μg of each sample was subjected to SWATH acquisition (34 swaths of 25 Da); two mass spectrometric technical replicates were acquired for each of the two biological replicates. To generate the spectral reference library, 2 μg of the pooled sample were subjected to traditional data-dependent acquisition (DDA). A library of 1967 proteins was created. After performing the extraction and integration processes using PeakView Software v2.2, 1796 proteins were exported into MarkerView 1.2.1 that together with GraphPad7.0 and Microsoft Excel 2010, was used for statistical analysis. BR: Biological Replicate; TR: Technical Replicate

**Fig. 2:**
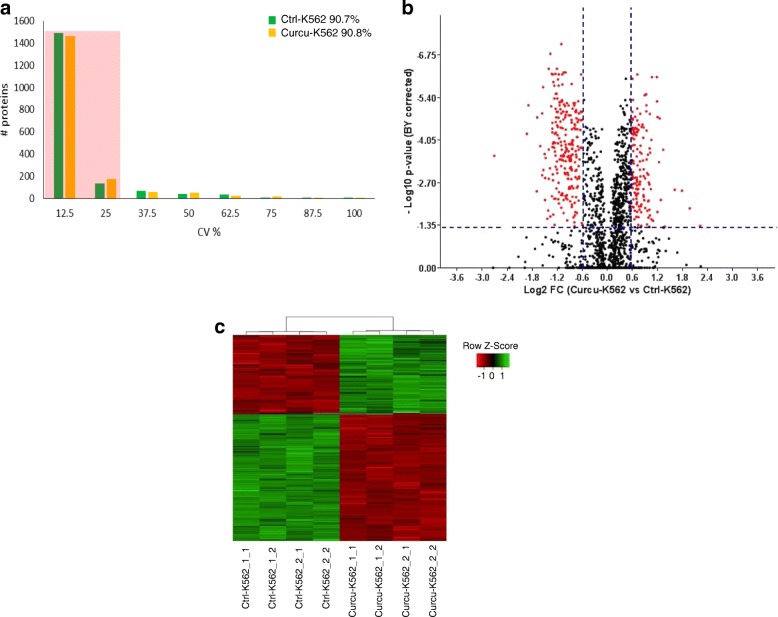
Statistical analysis of SWATH data. **a** Histogram shows the distribution of coefficients of variation (CVs) among technical and biological replicates of Ctrl-K562 and Curcu-K562 cells. More than 90% of the proteins as highlighted by the shadow have CV ≤ 25%. **b** Volcano plot of 1796 quantified proteins illustrating expression increases and decreases following curcumin treatment. Red dots correspond to proteins showing a fold change (FC) ≥ ±1.5 in relative abundance and a corrected BY *p*-value < 0.05 (values indicated by vertical and horizontal dashed lines) and considered significantly differentially modulated in untreated and curcumin-treated K562 cells. **c** Heat map analysis of 377 modulated proteins among technical and biological replicates of Ctrl-K562 and Curcu-K562 cells. The value of the MS signal intensity is shown

The aim of this study was to evaluate if within the first 24 h of treatment a sub-toxic dose of curcumin may induce significant biological effects in CML cells in order to propose the use of this natural compound as adjuvant of canonical anti-leukemic drugs. Based on preliminary data on cell survival obtained on K562 and LAMA84 cells treated with increasing doses of curcumin (Additional file 1: Figure S1a-b), we selected the dose of 20 μM of curcumin for all subsequent experiments performed within this study. The experimental design and workflow were based on application of SWATH label-free proteomics quantitative technology as illustrated in Fig. 1. A peptide mixture of both control (Ctrl-K562) and curcumin-treated K562 cells (Curcu-K562) was run using a Data-Dependent Acquisition (DDA) method in order to build the spectral library needed for the following SWATH-MS analysis. The integrated DDA data sets were searched against the *Homo sapiens* UniProt fasta database by using ProteinPilot 4.5 at a 1% critical false discovery rate (FDR), at both protein and peptide levels allowing the identification of 2059 proteins (the lists of identified peptides and proteins are shown in Additional file 2: Table S1, respectively in sheet “*Spectral reference library-pept*” and “*Spectral reference library-prot*”). More than 60% of the proteins were identified based on at least three peptides. The obtained spectral reference library was used for developing the following SWATH-MS strategy and 7852 targeted peptides (filtered using a FDR threshold of ≤5% over eight runs) allowed to obtain a detection rate of 75.3% (47,314 of 62,816) resulting in quantitative information for 1871 proteins (Additional file 3: Table S2, sheet “*Total protein quantification*”). The following comparative analysis was performed by using a filtered dataset of 1797 quantified proteins, in which deleted and uncharacterized proteins as well as proteins identified by only one peptide with high confidence but with less than 9 amino acids, were eliminated (Additional file 3: Table S2, sheet “*Filtered protein quantification*”). We found that among the technical and biological replicates, the percentage of proteins whose quantitation showed a coefficient of variation (CV) ≤ 25% in the quantitative data was 90.7% for the Ctrl-K562 group and 90.8% for Curcu-K562 group (Fig. 2a and Table 1). These results highlighted that the SWATH strategy used in this study ensures high throughput and reproducibility for protein quantitation. The robustness of the analysis was further confirmed by the analysis of variance showing that the R^2^ value for two repeats was never less than 0.99 in both the Ctrl-K562 group (Additional file 4: Figure S2) and Curcu-K562 group (Additional file 5: Figure S3). To identify differentially expressed proteins, a statistical analysis was applied, and proteins showing a fold change (FC) ≥ ±1.5 in relative abundance and a corrected BY *p*-value < 0.05 were considered differentially modulated in untreated and curcumin-treated K562 cells (Fig. 2b). In total, by comparing Curcu-K562 vs Ctrl-K562 we found 377 proteins significantly differentially expressed that were divided in two sets: the first consisted of 143 up-regulated proteins (Additional file 3: Table S2, sheet “*Curcu-UpRegProteins*”) and the second consisted of 234 down-regulated proteins (Additional file 3: Table S2, sheet “*Curcu-DownRegProteins*”). The intensity changes of the differentially expressed proteins are shown as heat map in Fig. 2c.

**Table 1:** Summary of protein identification results

Samples	Ctrl-K562				Curcu-K562			
	*Ctrl-K562_1*		*Ctrl-K562_2*		*Curcu-K562_1*		*Curcu-K562_2*	
	*Ctrl-K562_1_1*	*Ctrl-K562_1_2*	*Ctrl-K562_2_1*	*Ctrl-K562_2_2*	*Curcu-K562_1_1*	*Curcu-K562_1_2*	*Curcu-K562_2_1*	*Curcu-K562_2_2*
Numbers of proteins in DDA protein library	2061							
Numbers of peptides in DDA protein library	13616							
Numbers of proteins quantified in SWATH analysis	1801							
Numbers (and percentage) of quantified protein with CV ≤ 25% among technical and biological replicates	1629 (90.7%)				1632 (90.8%)			

### Bioinformatic analysis for enriched terms

**Fig. 3:**
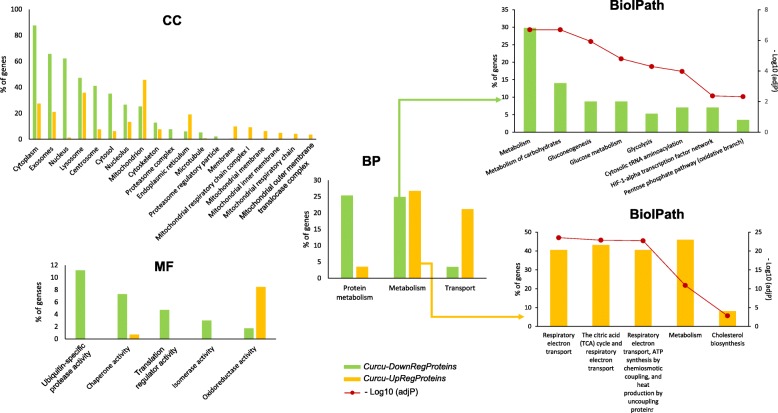
GO and Biological Pathway enrichment analysis of Curcu-Down-Regulated and Curcu-Up-Regulated proteins by FunRich. The histograms show for each GO term, Cellular Component (CC), Biological Process (BP) and Molecular Function (MF), the most significantly enriched categories (adjP ≤10–5; see Table 2) in the differentially expressed proteins (down−/up-regulated in K562 cells treated with curcumin). Biological Pathaway histograms (BiolPath) specify the functional categories of metabolic protein group enriched in both protein datasets (BP hisograms). Extended data of GO enrichment analysis is provided in Additional file 3: Table S2 - Curcu-DownReg Proteins_FunRichGOterms and Additional file 6: Table S3 - Curcu-UpReg Proteins_FunRichGOterms

To depict the functional classes significantly modulated in K562 cells treated with curcumin, the two sets of differentially expressed proteins were functionally categorized using the FunRich software, which performs a hypergeometric test for the enrichment of GO terms and pathways, followed by the Benjamini & Hochberg (BH) method for multiple test adjustment (adjP). The comparison between the 143 up-regulated proteins and the 234 down-regulated proteins provided interesting evidence about the molecular effects induced by curcumin treatment. Histograms in Fig. 3 and the Table 2 show the most significantly enriched GO categories (adjP ≤10^− 5^) for each of the three GO terms, Cellular Compartment (CC), Molecular Function (MF) and Biological Process (BP), in the sets of proteins down and up regulated in curcumin-treated K562 cells. Extended data of GO enrichment analysis is provided in Additional file 6: Table S3 and Additional file 7: Table S4.

**Table 2:** Most significantly enriched GO categories (adjP ≤10^−5^) within the differentially Curcu-regulated proteins datasets

GO term	# of proteins in the dataset ^(a)^	# of proteins in the background dataset ^(b)^	% of proteins	Fold enrichment	AdjP-Value (BH method)
Cellular Component (CC)					
*Enriched in Curcu-Down-Regulated protein dataset*					
Cytoplasm	204	5684	87.55	2.96	9.19E-75
Exosomes	153	2043	65.67	6.18	7.35E-89
Nucleus	145	5847	62.23	2.05	7.44E-22
Lysosome	110	1620	47.21	5.	8.46E-54
Centrosome	96	656	41.20	12.08	1.64E-76
Cytosol	82	1178	35.19	5.75	1.28E-38
Nucleolus	62	1257	26.61	4.07	3.99E-2
Mitochondrion	59	1259	25.32	3.87	5.61E-18
Cytoskeleton	30	427	12.88	5.8	6.42E-13
Proteasome complex	18	34	7.73	43.7	3.95E-24
Microtubule	12	129	5.15	7.68	3.70E-06
Proteasome regulatory particle	5	8	2.15	51.62	9.69E-07
*Enriched in Curcu-Up Regulated protein dataset*					
Mitochondrion	65	1259	45.77	6.99	1.91E-17
Lysosome	51	1620	35.92	4.26	3.69E-06
Endoplasmic reticulum	27	1104	19.01	3.31	2.89E-05
Mitochondrial respiratory chain complex I	13	46	9.15	38.29	2.39E-15
Mitochondrial membrane	9	56	6.34	21.78	3.73E-08
Mitochondrial inner membrane	7	58	4.93	16.36	2.22E-05
Mitochondrial respiratory chain	6	8	4.23	101.6	7.90E-10
Mitochondrial outer membrane translocase complex	5	6	3.52	112.88	1.91E-08
Molecular Function (MF)					
*Enriched in Curcu-Down-Regulated protein dataset*					
Ubiquitin-specific protease activity	26	377	11.16	5.69	9.80E-11
Chaperone activity	17	126	7.30	11.14	4.68E-11
Translation regulator activity	11	101	4.72	9	2.97E-06
Isomerase activity	7	45	3	12.85	6.02E-05
*Enriched in Curcu-Up Regulated protein dataset*					
Oxidoreductase activity	12	161	8.45	10.1	5.99E-07
Biological Process (BP)					
*Enriched in Curcu-Down Regulated protein dataset*					
Protein metabolism	59	1323	25.32	3.68	1.24E-16
Metabolism	58	1683	24.89	2.84	9.15E-12
*Enriched in Curcu-Up Regulated protein dataset*					
Metabolism	38	1683	26.76	3.06	2.22E-08
Transport	30	1215	21.13	3.34	2.32E-07

(a) The *Curcu-Up-Regulated protein dataset is composed by 233/234 proteins since the protein Q5HYB6_HUMAN (Gene Name: DKFZp686J1372)* was not mapped in FunRich database; the *Curcu-Up-Regulated protein dataset is composed by 142/143 proteins since the protein CD97_HUMAN (Gene name: CD97)* was not mapped in FunRich database; (b) The background dataset (in FunRich database) is composed by 19,230 genes/proteins

We found that most of the proteins down-regulated by curcumin treatment were associated with cytosolic localization (Fig. 3 histogram “CC”: Cytoplasm, Cytosol, Proteasome complex, Cytoskeleton, Microtubule, Proteasome regulatory particle. See Table 2 for the details of this analysis) and cytosolic functions (Fig. 3 histogram “MF”: Chaperone activity, Ubiquitin-specific protease activity, Translation regulator activity, Isomerase activity; Fig. 3 histogram “BP”: Protein metabolism. See Table 2 for the details of this analysis), while the group of proteins up-regulated by curcumin treatment were significantly enriched in the categories associated with mitochondrial localization (Fig. 3 histogram “CC”: Mitochondrion and 5 categories related to mitochondrial membrane localization. See Table 2 for the details of this analysis) and mitochondrial activities (Fig. 3 histogram “MF”: Oxidoreductase activity; Fig. 3 histogram “BP”: Transport. See Table 2 for the details of this analysis).

This divergence was further confirmed by analyzing the category “metabolism”, within the BP GO term. Indeed, even if this category was significantly enriched in both CurcuDown- and CurcuUp-Regulated Proteins set (Fig. 3 histogram “BP” and Table 2), the analysis of its specific composition showed the presences of functionally different proteins (as detailed in Additional file 6: Table S3 and Additional file 7: Table S4). As reported in BiolPath histograms in Fig. 3 and in Table 3, the Biological Pathway enrichment analysis revealed that the metabolic proteins down-regulated by curcumin were typically associated to glucose metabolism and that some of them were related to HIF-1α transcription factor network (ALDOA; PKM; LDHA; PGK1. See Table 3), whereas metabolic proteins up-regulated by curcumin were mostly related to mitochondrial activities.

**Table 3:** Biological Pathway enrichment analysis of “metabolic” proteins down-regulated and up-regulated by curcumin

Biological pathway	% of proteins	AdjP-Value (BH method)	Proteins
*Curcu-Down-Regulated “metabolic” proteins*			
Metabolism	29.82	2.04E-07	TALDO1; ARG2; GSTP1; AHCY; TXN; FASN; TKT; COX6B1; GMPS; PAICS; HPRT1; CAD; ATIC; GSTO1; ACLY; GOT1; TK1;
Metabolism of carbohydrates	14.04	2.04E-07	TPI1; TALDO1; GPI; MDH1; PGK1; TKT; PGD; GOT1;
Gluconeogenesis	8.77	1.19E-06	TPI1; GPI; MDH1; PGK1; GOT1;
Glucose metabolism	8.77	1.60E-05	TPI1; GPI; MDH1; PGK1; GOT1;
Glycolysis	5.26	5.13E-05	TPI1; GPI; PGK1;
Cytosolic tRNA aminoacylation	7.02	1.07E-04	DARS; PPA1; VARS; YARS;
HIF-1-alpha transcription factor network	7.02	4.28E-03	ALDOA; PKM; LDHA; PGK1;
Pentose phosphate pathway (oxidative branch)	3.51	4.78E-03	G6PD; PGD;
*Curcu-Up-Regulated “metabolic” proteins*			
Respiratory electron transport	40.5	3.0E-24	UQCRC1; CYC1; COX5B; NDUFS1; NDUFA5; NDUFB4; NDUFV1; UQCRH; NDUFA9; UQCRB; MT-ND5; NDUFB6; NDUFB5; NDUFB3; NDUFB9;
The citric acid (TCA) cycle and respiratory electron transport	43.2	1.4E-23	UQCRC1; CYC1; COX5B; NDUFS1; NDUFA5; NDUFB4; NDUFV1; UQCRH; NDUFA9; UQCRB; MT-ND5; NNT; NDUFB6; NDUFB5; NDUFB3; NDUFB9;
Respiratory electron transport, ATP synthesis by chemiosmotic coupling, and heat production by uncoupling proteins.	40.5	1.9E-23	UQCRC1; CYC1; COX5B; NDUFS1; NDUFA5; NDUFB4; NDUFV1; UQCRH; NDUFA9; UQCRB; MT-ND5; NDUFB6; NDUFB5; NDUFB3; NDUFB9;
Metabolism	45.9	1.4E-11	UQCRC1; CYC1; COX5B; NDUFS1; NDUFA5; NDUFB4; NDUFV1; CYP51A1; UQCRH; NDUFA9; UQCRB; MT-ND5; NNT; NDUFB6; NDUFB5; NDUFB3; NDUFB9;
Cholesterol biosynthesis	8.1	1.5E-03	FDFT1; CYP51A1; DHCR24;

For a deeper understanding of the biological meaning and relevance of effects induced in K562 cells by curcumin treatment, we performed functional enrichment analysis using ClueGO+CluePedia, a Cytoscape plug, which facilitates the visualization of functionally related genes/proteins displaying them as a clustered network [18]. The statistical test used for the enrichment analysis was based on right-sided hypergeometric option with a Benjamini-Hochberg correction, kappa score of 0.4 and a *p*-value < 0.05 was used as the cut-off criterion. The functional networks reported in Fig. 4 depict the results obtained by performing the enrichment analysis of KEGG pathway of CurcuUp-Regulated proteins (Fig. 4a) and Biological Process (BP) of CurcuDown-Regulated proteins (Fig. 4b). This analysis (detailed in Additional file 8: Table S5)*,* highlighted that within the group of CurcuUp-Regulated proteins seven GO Groups (nodes with the same color form a unique GO group) were enriched and among them two were constituted by multiple GO terms associated with mitochondrial structure and functions (Fig. 4a and Additional file 8: Table S5, sheet “*Curcu-UpRegProt_ClueGo*”). In contrast, we found that within the set of Curcu-down regulated proteins 14 GO groups were significantly enriched and 50% (7/14) were specifically associated and correlated to carbohydrate metabolism such as Pentose phosphate pathway, Glycolysis/Gluconeogenesis, Central carbon metabolism in cancer, Amino sugar and nucleotide sugar metabolism, Pyruvate metabolism, Cysteine and methionine metabolism and Purine metabolism (Fig. 4b and Additional file 8: Table S5, sheet “*Curcu-DownRegProt_ClueGo*”). Interestingly, some proteins, belonging to the Glycolysis/Gluconeogenesis GO group, were also indicated as specifically involved in the HIF-1α signaling pathways. Together, these results clearly demonstrate that curcumin-treatment induces alterations of metabolic activities characterizing CML cells and strongly related to the activity of HIF-1α pathway, known to have a critical role in in the pathogenesis of CML [4, 6, 7].

**Fig. 4:**
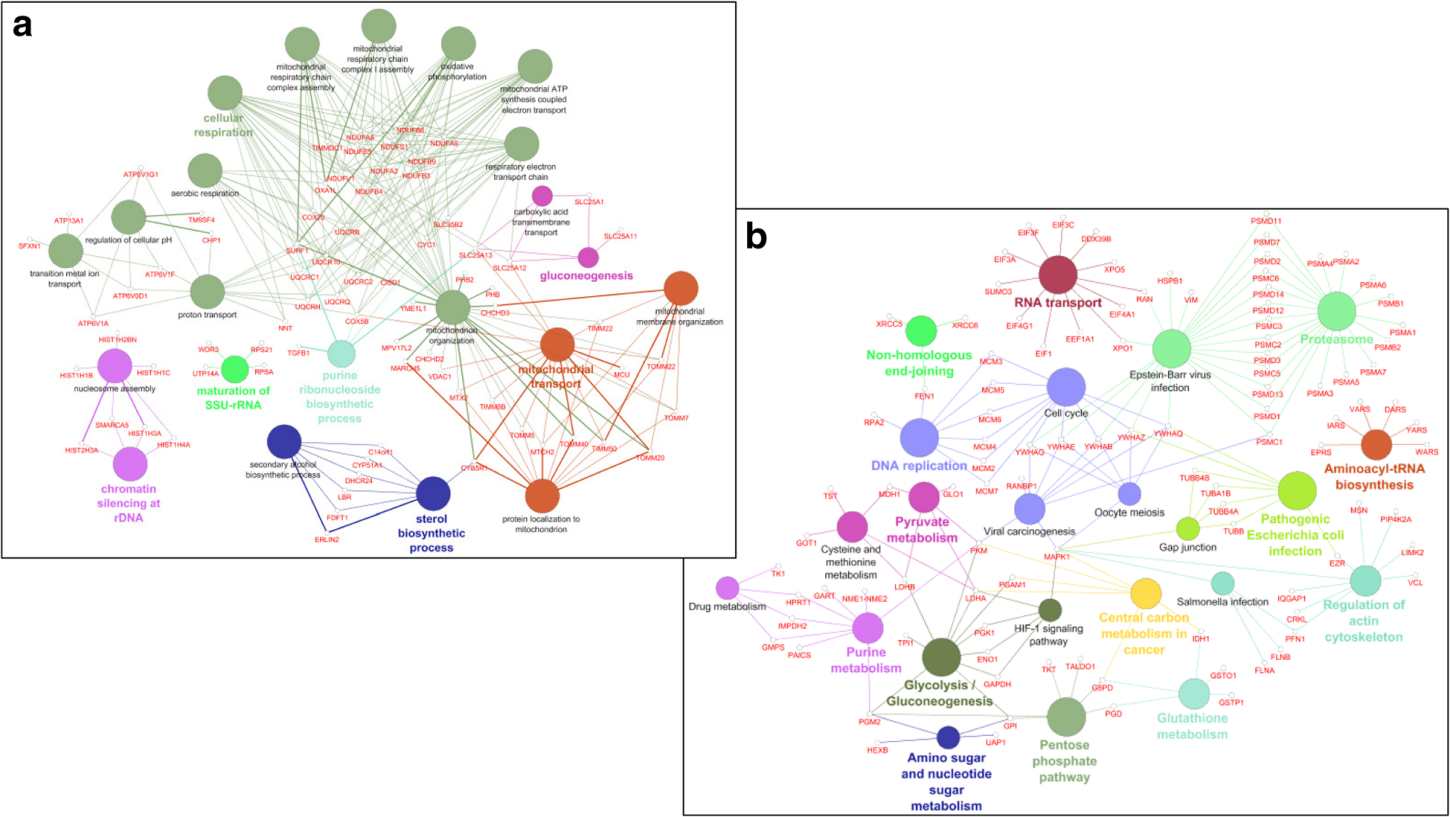
Enriched GO network groups using ClueGO/CluePedia-based enrichment. **a** Network view for KEGG pathway of CurcuUp-Regulated proteins and **b** Biological Process of CurcuDown-Regulated proteins. Terms (each represented as node) are functionally grouped based on shared genes (kappa score ≥ 0.4) and are shown with different colors. The specific players (proteins) of each node are highlighted with the respective gene name Node color represents the class that they belong. The size of the nodes indicates the degree of significance. Within each class, the most significant term (indicated with colored and bold characters) defines the name of the group. Ungrouped terms are not shown

### Effects of curcumin on HIF-1α activity and IPO7 expression

**Fig. 5:**
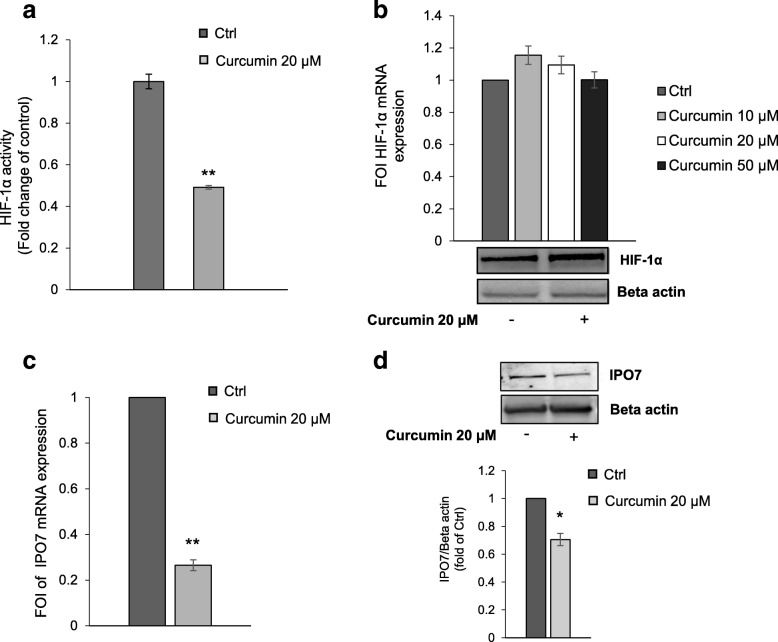
Effects of Curcumin on HIF-1α activity and IPO7 expression. **a** Assay of the transcriptional activity of HIF-1α showing that in K526 cells curcumin induced a reduction of HIF-1α activity compared to control cells. The reported values are the mean of three independent experiments. **b** qPCR (upper panel) and representative Western blot (lower panel) show that curcumin treatment did not affect HIF-1α at both mRNA and protein level. The values (FOI: Fold of Induction) in the histogram are normalized against GAPDH and are the mean ± SD of three independent experiments. **c** qPCR demonstrates that in K562 cells curcumin induced a decrease of mRNA IPO7 expression. The values (FOI: Fold of Induction) in the histogram are normalized to GAPDH and are the mean ± SD of three independent experiments. **d** Representative western blot and corresponding densitogram showing that curcumin inhibited the protein expression of IPO7 in K562 cells. In the Western blot assay, actin was used as loading control. Intensities of proteins bands were calculated from the peak area of densitogram by using Image J software. Ctrl: control cells. Statistical significance was calculated vs Ctrl: *p < 0.05, **p < 0.01

HIF-1α is the master regulator of cell response to hypoxic stress. However, it was reported that high levels of BCR-ABL expression correlate with a non-hypoxic induction of HIF-1α [7]. We found that in K562 cells curcumin elicited a significant decrease of HIF-1α activity (Fig. 5a) but without affecting its expression both at mRNA and protein level (Fig. 5b).

Transcription factors such as HIF-1α, to be functional and to activate transcription of target genes have to be imported from the cytoplasm to the nucleus through the nuclear pore complex (NPC). Importins, that are members of the β-karyopherin family, are nucleocytoplasmic factors that mediate nuclear import of proteins [24]. Intracellular distribution of HIF-1α requires a finely regulated balance between nuclear import and export. Consequently, the reduction of HIF-1α activity observed in K562 cells treated with curcumin, led us to hypothesize that curcumin may act by blocking the nuclear translocation of HIF-1α. Interestingly, in our protein dataset we found several proteins involved in nucleocytoplasmic transport (Table 4). Even if in cells treated with curcumin all these proteins showed a general down-regulation, based on the FC and corrected *p*-value cutoffs we used in this study, just four of these proteins could be considered significantly modulated by curcumin treatment: IPO5 (FC: -1.530; BY-Pvalue: 0.0383), IPO7 (FC: -2.332; BY-Pvalue: 0.0030); TNPO1 (FC: -1.801; BY-Pvalue: 0.0099), XPO1 (FC: -1.804; BY-Pvalue: 0.0026). In particular, it was described that importin 7 (IPO7), together with importin 4 (IPO4), is able to regulate the nuclear localization of HIF-1α [25]. Curcumin-induced IPO7 down-regulation highlighted by proteomic analysis was validated at both mRNA and protein level by real-time PCR (Fig. 5c) and Western blot (Fig. 5d) analyses. This data suggests that the mechanism through which the curcumin treatment induced in K562 cells a reduction of HIF-1α activity may be due to the reduction of its nuclear import rate.

**Table 4:** Proteins implicated in nuclear import/export identified by MS in K562cells

UniProt Accession Number	UniProt Entry Name	Gene Name	Protein Name	Fold Change *(Curcu-K562* vs *Ctrl K562)*	*p*-Value	BY-Pvalue
**O00410**	**IPO5_HUMAN**	**IPO5**	**Importin-5**	**-1.530**	**0.002162**	**0.0383**
**O95373**	**IPO7_HUMAN**	**IPO7**	**Importin-7**	**-2.332**	**0.000107**	**0.0030**
Q96P70	IPO9_HUMAN	IPO9	Importin-9	−2.241	0.121934	> 0.999
P52292	IMA1_HUMAN	KPNA2	Importin subunit alpha-1	−1.040	0.040815	0.5065
O00629	IMA3_HUMAN	KPNA4	Importin subunit alpha-3	−1.279	0.002692	0.0465
O00505	IMA4_HUMAN	KPNA3	Importin subunit alpha-4	−1.667	0.086100	0.9772
Q14974	IMB1_HUMAN	KPNB1	Importin subunit beta-1	−1.114	0.000088	0.0026
**Q92973**	**TNPO1_HUMAN**	**TNPO1**	**Transportin-1**	**−1.801**	**0.00045**	**0.0099**
E9PFH4	E9PFH4_HUMAN	TNPO3	Transportin-3	−1.875	0.221323	> 0.999
**O14980**	**XPO1_HUMAN**	**XPO1**	**Exportin-1**	**−1.804**	**0.000086**	**0.0026**
P55060	XPO2_HUMAN	CSE1L	Exportin-2	−1.530	0.011327	0.1659
Q9HAV4	XPO5_HUMAN	XPO5	Exportin-5	**−1.698**	**0.000021**	**0.0008**
P12270	TPR_HUMAN	TPR	Nucleoprotein TPR	−1.121	0.001823	0.0330
Q9UKX7	NUP50_HUMAN	NUP50	Nuclear pore complex protein Nup50	1.334	0.246	> 0.999

Proteins considered significantly modulated by curcumin treatment (Fold Change ≥1.5 and BY-Pvalue ≤10^−5^) are in bold

Analogous effects of curcumin treatment on HIF-1α expression and activity as well as on IPO7 expression were also observed in LAMA84 cells (Additional file 9: Figure S4a-d).

Interestingly, we found that in both K562 and LAMA84 cells the down-regulation of HIF-1α activity induced by curcumin coincided with its reduced nuclear localization (Additional file 10: Figure S5).

### Curcumin inhibits IPO7 expression through the activation of miR-22

**Fig. 6:**
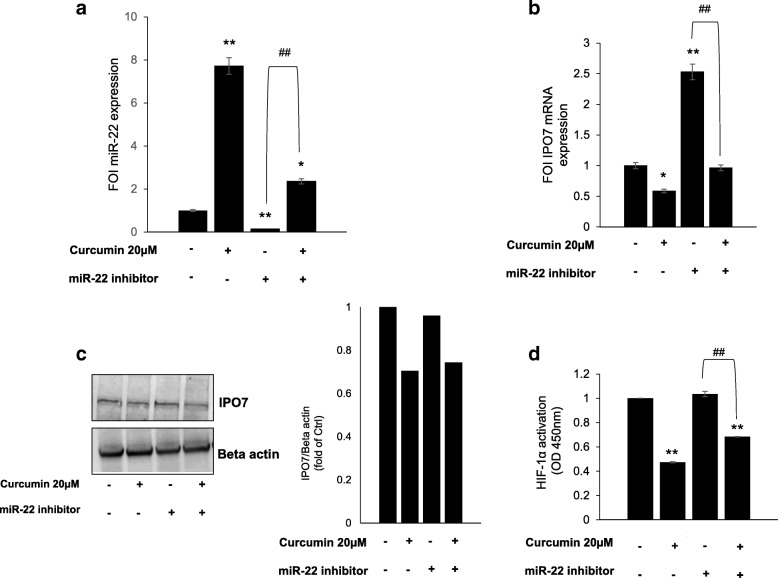
Inhibition of IPO7 expression induced by curcumin is miR-22 mediated. **a** qRT-PCR showing the ability of curcumin to induce in K562 cells a significant increase of miR-22 expression. Ctrl: control. The values (FOI: Fold of Induction) in the histogram are normalized against RNU6–2 and are the mean ± SD of three independent experiments. **b** qPCR shows that in K562 cells the inhibitory effect on IPO7 mRNA expression induced by miR-22 inhibitor transfection was reverted by curcumin treatment. The values (FOI: Fold of Induction) in the histogram are normalized against GAPDH and are the mean ± SD of three independent experiments. **c** Representative western blot and corresponding densitogram showing that in K562 cells the inhibitory effect on IPO7 protein expression induced by miR-22 inhibitor transfection was reverted by curcumin treatment. Actin was used as loading control. Intensities of proteins bands were calculated from the peak area of densitogram by using Image J software. **d** Assay of the transcriptional activity of HIF-1α showing that in K562 cells transfected with miR-22 inhibitor HIF-1α activity was significantly decreased when cells were co-treated with curcumin. Ctrl: control cells. Statistical significance was calculated vs Ctrl (*p < 0.05, **p < 0.01) or as indicated by line (##*p* < 0.01)

Since several studies showed that curcumin exerts its anti-cancer effect by regulating the expression of microRNAs (miRs) [26], we utilized miR target prediction software miRSearch V3.0 [27] to determine if IPO7 is subject to regulation by miRs. This analysis showed that IPO7 is a validated target of miR-22 and miR-9 [28, 29] (Additional file 11: Figure S6a). In particular we focused our attention on miR-22 since further analysis of predicted multiple targets performed by MicroRNA Target prediction (miRTar) tool (http://mirtar.mbc.nctu.edu.tw/human/) revealed that, within the dataset of CurcuDown-Regulated proteins beside IPO7 there were several miR-22 targets (Additional file 11: Figure S6b), while no targets of miR-9 were found. Moreover, a direct ability of curcumin to up-regulate miRNA-22 expression has been described [30–33]. To evaluate whether in our cell model miR-22 was modulated by curcumin treatment, we performed a qRT- PCR on control and curcumin-treated K562 cells. We found that in cells treated with curcumin, miR-22 was about 10-fold up-regulated in comparison to control cells and the reduction of miR-22 expression in K562 cells transfected with miR-22 inhibitor was recovered when cells were simultaneously treated with curcumin (Fig. 6a). According to this result, we also observed that the expression of IPO7 (at both mRNA and protein level) observed in K562 cells transfected with miR-22 inhibitor, was significantly reduced when cells were contemporarily treated with curcumin (Fig. 6b and c). Finally, in order to test if the axis miR-22/IPO7 could be correlated with the decrease of HIF-1α activity induced by curcumin, we performed a HIF-1α activity assay in K562 cells transfected with miR-22 inhibitor treated or not with curcumin. We found that the level of HIF-1α activity observed in K562 cells transfected with miR-22 inhibitor (comparable to the control), was significantly decreased when cells were in parallel treated with curcumin, suggesting that curcumin was able to counteract the miR-22 inhibition (Fig. 6d).

Analogous effect of curcumin treatment on miR-22 expression was observed in LAMA84 cells (Additional file 9: Figure S4e).

### Curcumin enhances the sensitivity of K562 cells to imatinib

**Fig. 7:**
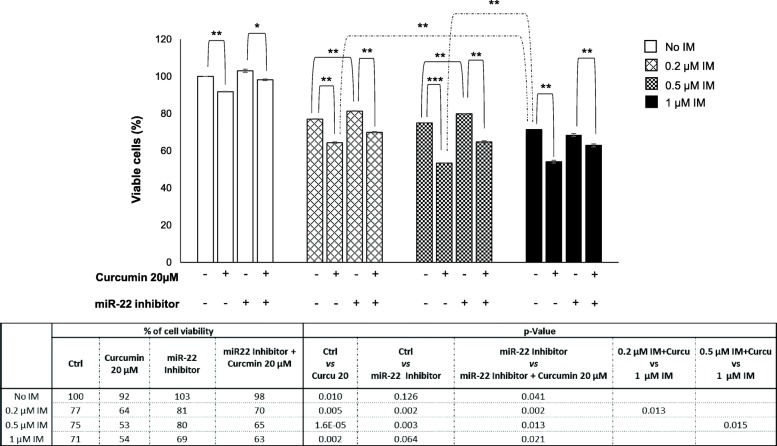
Curcumin induces a miR-22-mediated enhancement of anti-proliferative effects of imatinib. Cell viability assay (MTT assay) of K562 cells treated as indicated for 24 h, shows that curcumin induced a miR-22-mediated sensitization to imatinib. The reported values are the mean of three independent experiments; statistical significance was calculated as specified by lines: *p < 0.05, **p < 0.01. The table reports in detail the values of the histogram. Ctrl: cells in DMSO or with increasing doses of only imatinib; IM: imatinib

In order to assess whether curcumin-induced modulation of miR-22 was able to impact on K562 cells sensitivity to imatinib (IM), we evaluated the cell viability by treating cells for 24 h with curcumin and miR-22 inhibitor alone or in co-treatment with increasing doses of IM. As indicated in Fig. 7, curcumin that already alone induces a significant cell viability reduction of about 10%, is clearly able to increase the IM effectiveness for each of tested dose. In both histogram and table in Fig. 7 it is possible to observe that the addition of curcumin to IM treatment, induced a significant decrease of cell viability compared to treatment with IM alone. To note that compared to the effect of 1 μM of IM alone (75% of cell viability) both doses of 0.2 μM and 0.5 μM IM in co-treatment with curcumin elicited a significantly stronger negative effect on cell growth (respectively 64% - *p*-value = 0.013 and 53% - *p*-value = 0.015; Fig. 7). Interestingly, we noted in K562 cells transfected with miR-22 a low-level but significant resistance to 0.2 μM and 0.5 μM IM that was completely reverted when cells were co-treated with curcumin (Fig. 7). This data confirmed our hypothesis that curcumin effects are miR-22-mediated. Moreover, combination index (CI) analysis [22, 23] assessed by using a non-constant ratio of curcumin and imatinib (Additional file 12: Figure S7) showed that curcumin and imatinib have a synergistic effect on inhibition of both K562 and LAMA cell proliferation. CI results showed synergism especially when curcumin was used with lower doses of imatinb.

## Discussion

Nutraceuticals play an increasingly important role in the cancer management [8]. Among them curcumin, a natural polyphenol extracted from *Curcuma longa* L., has been shown to have pleotropic anti-cancer properties (anti-oxidant, anti-inflammatory, anti-proliferative) inhibiting multiple targets and molecular pathways such as NF-kB, STAT3, MAPK, PTEN, P53, AKT/mTOR, VEGF and microRNAs (miRNA) network involved in cancer pathogenesis [9, 11, 34, 35]. Data accumulated in the last years has revealed that curcumin is a promising pharmacologically safe anti-tumor agent that can function as chemosensitizer and a multi-targeted inhibitor. However, actually the use of curcumin as therapeutic remedy is strongly limited by its extremely low solubility in water and in organ fluids, which subsequently limits the bioavailability and therapeutic effects of curcumin. Currently, continuous attempts are going to improve solubilization and bioavailability of this promising agent. For example it has been demonstrated that heat and pressure can significantly improve solubility of curcumin maintaining its therapeutic properties [36–39].

The benefits of curcumin have been demonstrated also in blood cancer. In acute lymphoblastic leukemia Philadelphia chromosome-positive (Ph + ALL), it was demonstrated that curcumin potentiated the efficiency of imatinib by inhibiting the activation of the AKT/mTOR signaling and by down-regulating the expression of the BCR/ABL gene [40]. Similarly in CML it was demonstrated that curcumin treatment induced the decrease of BCR-ABL expression at both mRNA and protein level associated to a dose-dependent increase of PTEN and a decreased AKT phosphorylation and VEGF expression due to a selective packaging of miR-21 in exosomes [11].

In the present study, for the first time, we demonstrated that in CML cells curcumin at sub toxic dose affects the HIF-1α pathway that it is known to promote leukemic cell proliferation and to play a key role in the progressive loss of sensitivity to imatinib [5–7].

We have applied SWATH-MS (sequential window acquisition of all theoretical spectra-mass spectrometry)-based quantitative proteomic analysis to examine the effect of curcumin on proteome of K562 cells, that our previous data indicated to have an intrinsic resistance to imatinib, likely related to the expression of several proteins implicated in drug resistance and with anti-apoptotic activity [41]. We found that in CML cells curcumin induced, compared to the untreated condition, a remarkable down-regulation of proteasome activity, as described in literature [42, 43], and of proteins associated to carbohydrate metabolism. In particular, we found that several enzymes involved in glycolysis/gluconeogenesis (TPI1; GPI; MDH1; PGK1; GOT1), in oxidative branch of pentose phosphate pathway (G6PD; PGD) and some direct targets of HIF-1α (ALDOA; PKM; LDHA; PGK1) were down-regulated in response to curcumin treatment. The dependence of cancer cells on glycolysis process for energy production rather than oxidative phosphorylation, is so frequent and common in majority, if not, all types of tumors, to be considered as a molecular signature of cancer [44–46]. This tumor glycolysis, associated to more aggressive tumor phenotype, is directly controlled by major signal transduction pathways involved in oncogenesis often associated with HIF-1α activity [47, 48]. In CML cells, in particular, increased glycolytic activity has been found to depend on the non-hypoxic activation of HIF-1α and has been described as marker for early detection of imatinib resistance, before clinical manifestations [7, 49]. Moreover, data from literature has provided strong evidence in support of the crucial role of HIF-1α in the pathogenesis of CML, by promoting cell proliferation and survival maintenance of LSCs [5–7]. Thus, the co-suppression of BCR-ABL and HIF-1α can offer the opportunity to develop a rational therapeutic strategy for CML eradication.

Consistent with the obtained proteomic data evidencing the down-regulation of several HIF-1α targets and associated pathways, we found that in K562 cells curcumin induced a decrease of HIF-1α activity. Previous studies have demonstrated that the anti-cancer properties of curcumin may be related to its ability to affect the HIF-1 pathway, affecting the expression of HIF-1α or by degrading ARNT (HIF1β) but without altering the expression and the transcriptional activity of HIF-1α [50, 51]. Interestingly, in the present study we found that in CML cells curcumin induced a significant inhibition of HIF-1α activity, without affecting its expression. These observations led us to hypothesize that curcumin could have an indirect effect on HIF-1α.

Data from literature reported that curcumin is able to affect nuclear traffic by inhibiting CRM1 (exportin1 or Xpo1) [52], that we also found down-regulated in curcumin-treated K562 cells together with other proteins implicated in nuclear import/export (in total 14 of these proteins were identified in our dataset). Interestingly among them there was IPO7, an importin specifically related to HIF-1α nuclear translocation [25]. According to literature data strongly supporting the ability of curcumin to regulate miRNA expression [11, 26, 31, 33, 34], we found that in K562 cells the curcumin-dependent down-regulation of IPO7 was miR-22-mediated. To deeper define the molecular steps through which the HIF-1α/miR22/IPO7 axis mediates the curcumin effects on CML cells future research can be aimed to define the timing in which HIF-1α nuclear localization is modulated as well as to identify other interactors acting within this system.

Evidence accumulated in recent years has defined the key role of nuclear transport in regulating the features of cancer cells and highlighted the potential role of nuclear transport proteins as new therapeutic targets for developing combination treatment strategies [24]. For example, the ability of XPO1 inhibitors to enhance the therapeutic effects of some available chemotherapeutic agents used for treating both haematological and solid tumours was reported [53–55]. Interestingly, we found that through its miR-22-mediated activity, curcumin on K562 cells enhanced the anti-proliferative effects of imatinib. This finding adds new evidence about the molecular mechanisms through which curcumin can enhance the efficacy of anti-cancer drugs working as chemosensitizer [43, 56, 57]. From a clinical point of view this is an interesting data since it is widely recognized that during treatment with imatinib, dose optimization proved an effective tool to reach adequate response by improving efficacy and reducing toxicity and costs of treatment [58].

## Conclusions

**Fig. 8:**
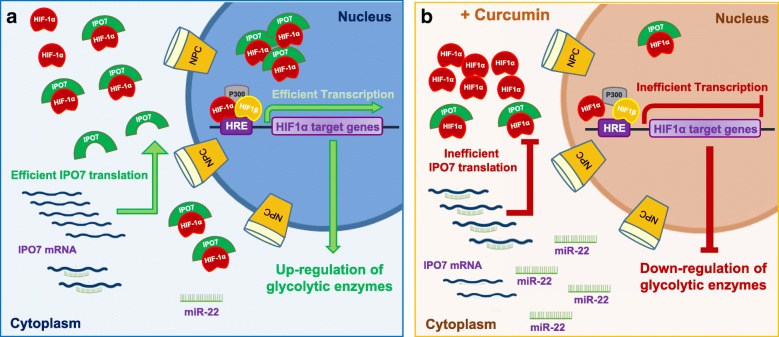
The proposed model of miR-22/IPO7/HIF-1a axis modulation induced by curcumin in CML. **a** In basal condition CML cells are characterized by an efficient HIF-1α activity responsible of active transcription of several target genes some of which associated to glycolytic metabolism. **b** Curcumin by inducing up-regulation of miR-22 elicits the inhibition of IPO7 expression, hindering the HIF-1α translocation and causing the global down-regulation of glycolytic enzymes as highlighted by our quantitative proteomic data. NCP: Nuclear Pore Complex

In conclusion, our data highlighted that in CML cells curcumin, beyond its well-known ability to inhibit the proteasome pathway [42, 43], activates alternative molecular networks affecting the glycolytic metabolism that in CML is due to non-hypoxic activation of HIF-1α [7, 49]. We depicted a new molecular scenario in which curcumin, by up-regulating miR-22 expression level, elicits the decrease of IPO7 and consequently hinders the nuclear translocation of HIF-1α essential for its activity, thus affecting the metabolic enzyme profile of CML cells (Fig. 8).

Our study, for the first time, suggests the miR-22/IPO7/HIF-1α axis as new molecular target of curcumin, revealing innovative therapeutic implications of this natural compound for treatment of CML as well as of other cancer types in which HIF-1α has a prominent role.

## Additional files

Additional file 1: **Figure S1.** Cell growth was measured by MTT assay after 24 h of treatment with increasing doses of curcumin. Each point represents the mean ± SD of three independent experiments. * ≤ 0.05. (PPTX 42 kb)

Additional file 2: **Table S1.** MS Data of Protein ID. (XLSX 2197 kb)

Additional file 3: **Table S2.** SWATH-MS Data. (XLSX 884 kb)

Additional file 4: **Figure S2.** Pearson’s R2 showing the correlation between biological and technical replicates of Ctrl-K562 cells. (PPTX 178 kb)

Additional file 5: **Figure S3.** Pearson’s R2 showing the correlation between biological and technical replicates of Curcu-K562 cells. (PPTX 185 kb)

Additional file 6: **Table S3.** DownReg Proteins_FunRichGOterms. (XLSX 50 kb)

Additional file 7: **Table S4.** UpReg Proteins_FunRichGOterms. (XLSX 35 kb)

Additional file 8: **Table S5.** Regulated Proteins_ClueGO Results. (XLSX 22 kb)

Additional file 9: **Figure S4.** Effects of Curcumin on HIF-1α activity, IPO7 expression and miR22 expression in LAMA84 cells. a Assay of the transcriptional activity of HIF-1α showing that in LAMA84 cells curcumin induced a reduction of HIF-1α activity compared to control cells. The reported values are the mean of three independent experiments. b qPCR (left panel) and representative Western blot (right panel) show that in LAMA84 cells curcumin treatment did not affect HIF-1α at both mRNA and protein level. The values (FOI: Fold of Induction) in the histogram are normalized against GAPDH and are the mean ± SD of three independent experiments. c qPCR demonstrates that in LAMA84 cells curcumin induced a decrease of mRNA IPO7 expression. The values (FOI: Fold of Induction) in the histogram are normalized to GAPDH and are the mean ± SD of three independent experiments. d Representative western blot and corresponding densitogram showing that in LAMA84 cells curcumin inhibited the protein expression of IPO7. e qRT-PCR showing the ability of curcumin to induce in LAMA84 cells a significant increase of miR-22 expression. The values (FOI: Fold of Induction) in the histogram are normalized against RNU6–2 and are the mean ± SD of two independent experiments. In the Western blot assay, actin was used as loading control. Intensities of proteins bands were calculated from the peak area of densitogram by using Image J software. Ctrl: control cells. Statistical significance was calculated vs Ctrl: *p < 0.05, **p < 0.01. (PPTX 732 kb)

Additional file 10: **Figure S5.** Representative western blots and corresponding densitograms showing that in K562 (a) and LAMA84 cells (b) curcumin decreased nuclear levels of HIF-1α. Ponceau S of nuclear extract was used as loading control. Intensities of proteins band (in Ponceau S the band used is indicated with arrow) were calculated from the peak area of densitogram by using Image J software. Ctrl: control cells. (PPTX 809 kb)

Additional file 11: **Figure S6.** IPO7/miRNAs correlation. a Analysis performed by using microRNA target prediction software miRSearch V3.0 showed that IPO7 is a validated target of miR-22 and miR-9. b Analysis of predicted multiple targets performed by MicroRNA Target prediction (miRTar) tool (http://mirtar.mbc.nctu.edu.tw/human/) revealed within the CurcuDown-Regulated dataset the presence of several of miR-22 targets beside IPO7. No target of miR-9 was found. (PPTX 179 kb)

Additional File 12: **Figure S7.** Anti-proliferative effects of curcumin, imatinib and curcumin+imatinib combination on CML cell viability. Curcumin and imatinib were tested for their anti-proliferative effects on K562 (a) and LAMA84 cells (b). The assays were performed by using curcumin and imatinib singly (using the reported doses) or in combination (20 μM curcumin held constant and imatinib at reported concentrations. In K562 cells combination compound treatments showed significant differences compared to single imatinib treatments for all doses tested (*p* < 0.001). In LAMA84 cells combination compound treatments showed significant differences compared to single imatinib treatments for lower doses tested (p < 0.001 at 0.1 and 0.2 μM), while no significant differences were observed between combination compound and imatinib at 0.5–5 μM because high cell death occurred. Combination Index (CI) analysis of growth inhibition in K562 (c) and LAMA84 cells (d) after 48 h incubation using curcumin (20 μM) and imatinib (different concentrations). Data from Fig. S6a and S6b were converted to Fraction Affected (FrAf) and plotted against Combination Index (CI). Results were as follows for imatinib concentration: ▲ = 0.1 μM; ♦ = 0.2 μM; ● = 0.5 μM; □ = 1 μM; ○ = 5 μM. Straight line on the graph designates a CI equal to 1. Combination Index interpretation was as follows: CI value of 1 indicates additivity; CI < 1 indicates synergism; and CI > 1 indicates antagonism. (PPTX 50 kb)
