# Supplementary material for: SWATH-MS based quantitative proteomics analysis reveals that curcumin alters the metabolic enzyme profile of CML cells by affecting the activity of miR-22/IPO7/HIF-1α axis
Source: J Exp Clin Cancer Res. 2018 Jul 25;37:170. doi: 10.1186/s13046-018-0843-y (PMC6060558; doi:10.1186/s13046-018-0843-y)
Supplement: Supplementary file 1 — Figure S1. Cell growth was measured by MTT assay after 24 h of treatment with increasing doses of curcumin. Each point represents the mean ± SD of three independent experiments. * ≤ 0.05. (PPTX 42 kb) [file 13046_2018_843_MOESM1_ESM.pptx]

## Slide 1
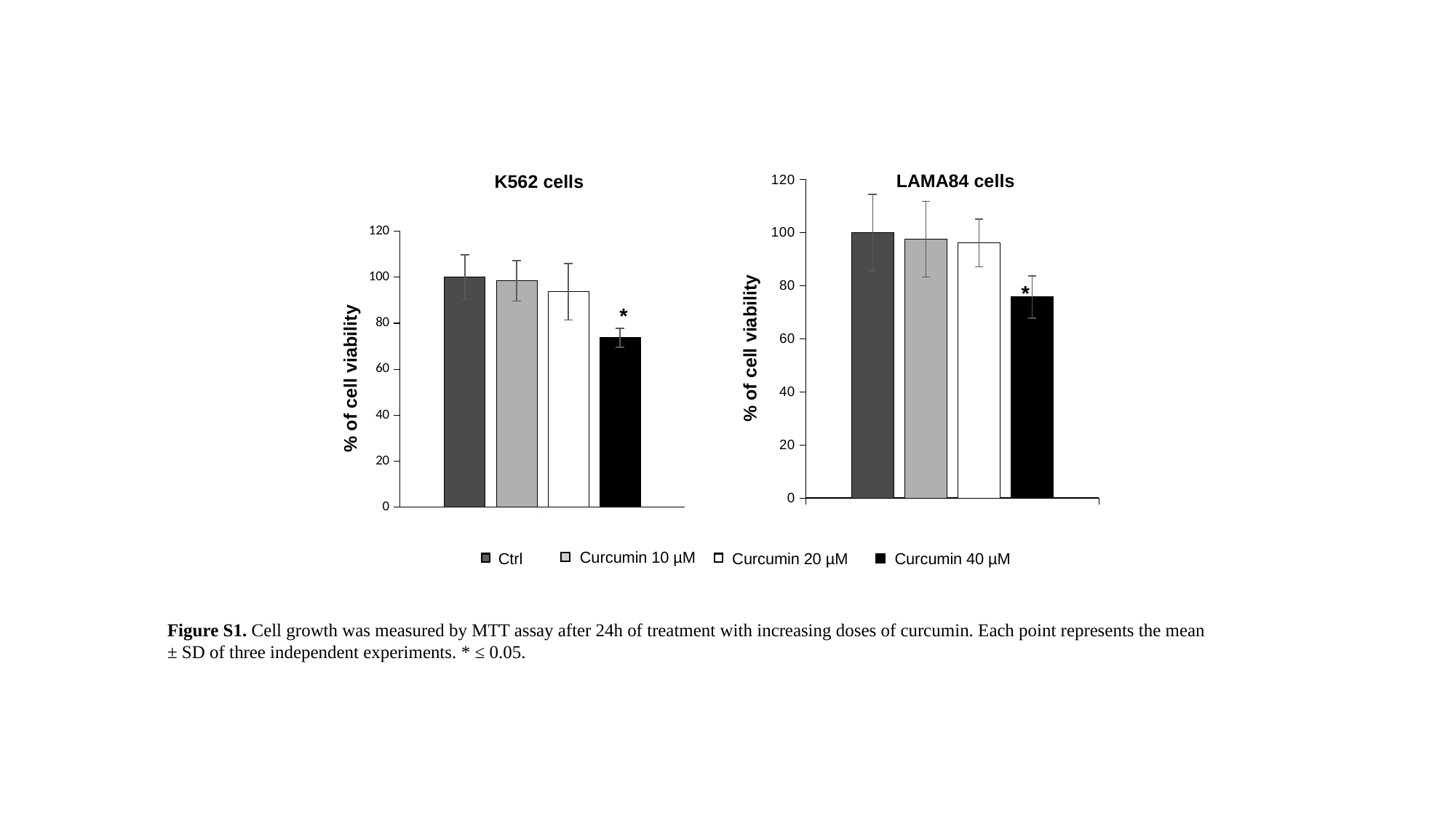

LAMA84 cells
### Chart
| Category | Ctrl | Curcu 10 µM | Curcu 20 µM | curcu 40 µM |
|---|---|---|---|---|*
% of cell viability
K562 cells
### Chart
| Category | Ctrl | Curcu 10 µM | Curcu 20 µM | curcu 40 µM |
|---|---|---|---|---|
*
% of cell viability
Curcumin 10 µM
Ctrl
Curcumin 20 µM
Curcumin 40 µM
Figure S1. Cell growth was measured by MTT assay after 24h of treatment with increasing doses of curcumin. Each point represents the mean ± SD of three independent experiments. * ≤ 0.05.
