# Supplementary material for: SWATH-MS based quantitative proteomics analysis reveals that curcumin alters the metabolic enzyme profile of CML cells by affecting the activity of miR-22/IPO7/HIF-1α axis
Source: J Exp Clin Cancer Res. 2018 Jul 25;37:170. doi: 10.1186/s13046-018-0843-y (PMC6060558; doi:10.1186/s13046-018-0843-y)
Supplement: Supplementary file 4 — Figure S2. Pearson’s R2 showing the correlation between biological and technical replicates of Ctrl-K562 cells. (PPTX 178 kb) [file 13046_2018_843_MOESM4_ESM.pptx]

## Slide 1
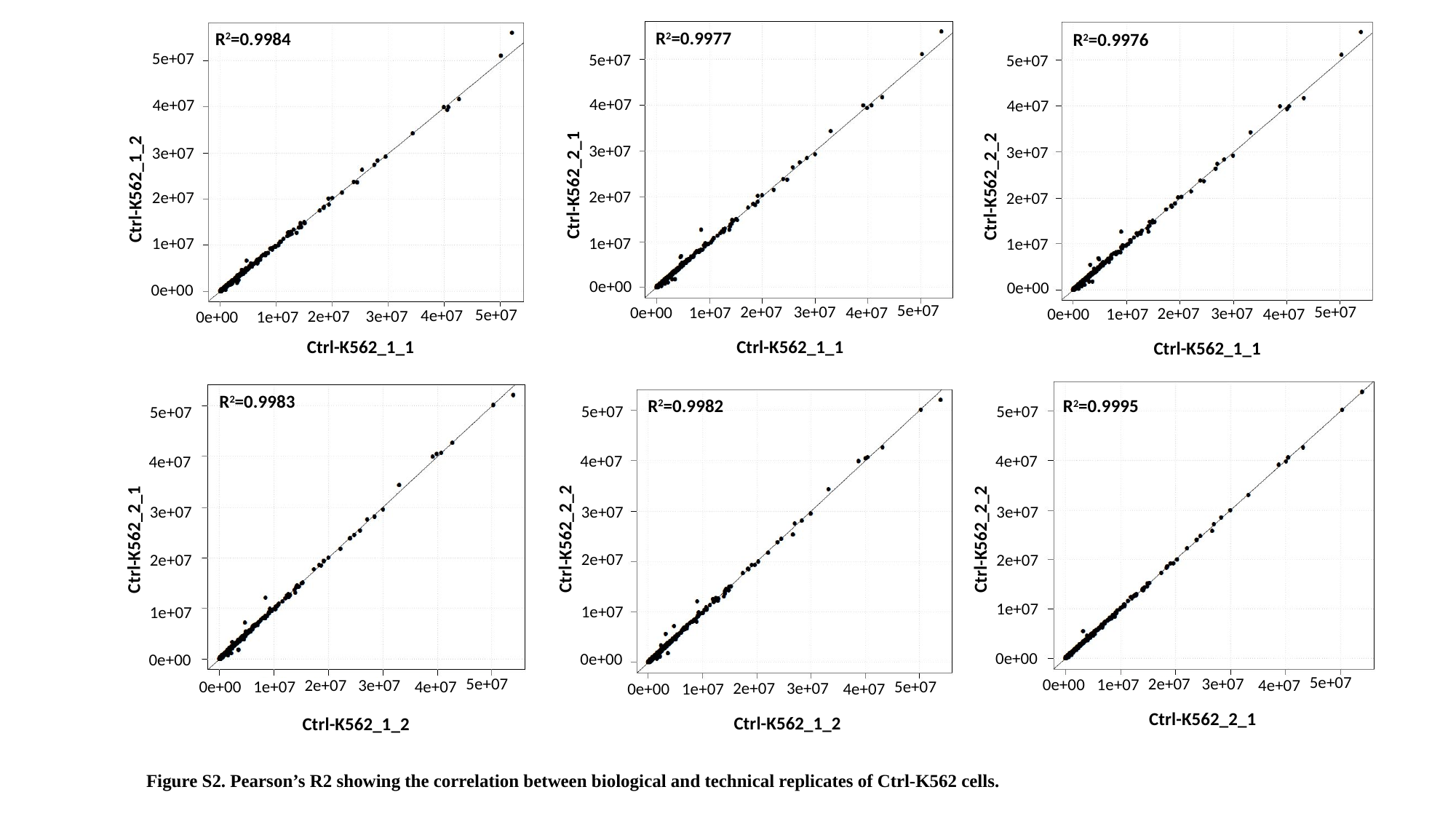

R2=0.9976
5e+07
4e+07
3e+07
2e+07
1e+07
0e+00
Ctrl-K562_2_2
Ctrl-K562_1_1
R2=0.9977
5e+07
4e+07
3e+07
2e+07
1e+07
0e+00
5e+07
2e+07
3e+07
1e+07
0e+00
4e+07
Ctrl-K562_1_1
R2=0.9984
5e+07
4e+07
3e+07
2e+07
1e+07
0e+00
Ctrl-K562_1_2
5e+07
4e+07
2e+07
3e+07
1e+07
0e+00
Ctrl-K562_1_1
Ctrl-K562_2_1
5e+07
2e+07
3e+07
1e+07
0e+00
4e+07
R2=0.9995
5e+07
4e+07
3e+07
2e+07
1e+07
0e+00
Ctrl-K562_2_2
5e+07
2e+07
3e+07
1e+07
0e+00
4e+07
Ctrl-K562_2_1
R2=0.9983
5e+07
4e+07
3e+07
2e+07
1e+07
0e+00
Ctrl-K562_2_1
5e+07
2e+07
3e+07
1e+07
0e+00
4e+07
Ctrl-K562_1_2
R2=0.9982
5e+07
4e+07
3e+07
2e+07
1e+07
0e+00
Ctrl-K562_2_2
5e+07
2e+07
3e+07
1e+07
0e+00
4e+07
Ctrl-K562_1_2
Figure S2. Pearson’s R2 showing the correlation between biological and technical replicates of Ctrl-K562 cells.
