# Supplementary material for: SWATH-MS based quantitative proteomics analysis reveals that curcumin alters the metabolic enzyme profile of CML cells by affecting the activity of miR-22/IPO7/HIF-1α axis
Source: J Exp Clin Cancer Res. 2018 Jul 25;37:170. doi: 10.1186/s13046-018-0843-y (PMC6060558; doi:10.1186/s13046-018-0843-y)
Supplement: Supplementary file 9 — Figure S4. Effects of Curcumin on HIF-1α activity, IPO7 expression and miR22 expression in LAMA84 cells. a Assay of the transcriptional activity of HIF-1α showing that in LAMA84 cells curcumin induced a reduction of HIF-1α activity compared to control cells. The reported values are the mean of three independent experiments. b qPCR (left panel) and representative Western blot (right panel) show that in LAMA84 cells curcumin treatment did not affect HIF-1α at both mRNA and protein level. The values (FOI: Fold of Induction) in the histogram are normalized against GAPDH and are the mean ± SD of three independent experiments. c qPCR demonstrates that in LAMA84 cells curcumin induced a decrease of mRNA IPO7 expression. The values (FOI: Fold of Induction) in the histogram are normalized to GAPDH and are the mean ± SD of three independent experiments. d Representative western blot and corresponding densitogram showing that in LAMA84 cells curcumin inhibited the protein expression of IPO7. e qRT-PCR showing the ability of curcumin to induce in LAMA84 cells a significant increase of miR-22 expression. The values (FOI: Fold of Induction) in the histogram are normalized against RNU6–2 and are the mean ± SD of two independent experiments. In the Western blot assay, actin was used as loading control. Intensities of proteins bands were calculated from the peak area of densitogram by using Image J software. Ctrl: control cells. Statistical significance was calculated vs Ctrl: *p < 0.05, **p < 0.01. (PPTX 732 kb) [file 13046_2018_843_MOESM9_ESM.pptx]

## Slide 1
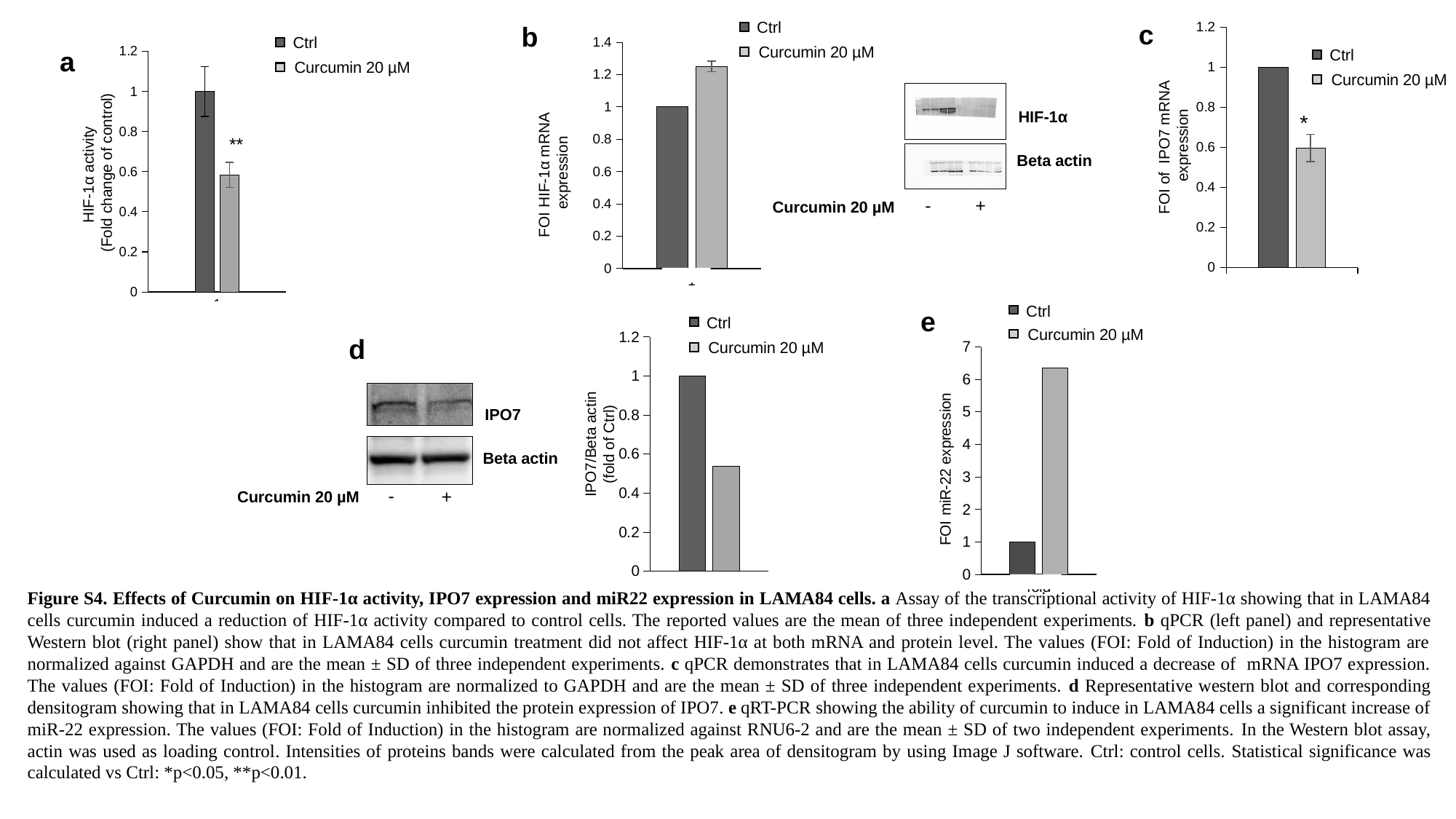

c
### Chart
| Category | Ctrl | Curcumin 20 µM |
|---|---|---|FOI of IPO7 mRNA
 expression
*
Ctrl
Curcumin 20 µM
Ctrl
Curcumin 20 µM
b
### Chart
| Category | Ctrl | Curcu 20 µM |
|---|---|---|
HIF-1α
Beta actin
-
+
Curcumin 20 µM
FOI HIF-1α mRNA
 expression
Ctrl
Curcumin 20 µM
### Chart
| Category | Ctrl | +curcu |
|---|---|---|HIF-1α activity
 (Fold change of control)
a
**
Ctrl
Curcumin 20 µM
e
### Chart
| Category | Ctrl | Curcu 20 µM |
|---|---|---|
| fold | 1.0 | 6.351952343593008 |
Ctrl
Curcumin 20 µM
### Chart
| Category | | |
|---|---|---|IPO7/Beta actin
(fold of Ctrl)
d
IPO7
Beta actin
-
+
Curcumin 20 µM
Figure S4. Effects of Curcumin on HIF-1α activity, IPO7 expression and miR22 expression in LAMA84 cells. a Assay of the transcriptional activity of HIF-1α showing that in LAMA84 cells curcumin induced a reduction of HIF-1α activity compared to control cells. The reported values are the mean of three independent experiments. b qPCR (left panel) and representative Western blot (right panel) show that in LAMA84 cells curcumin treatment did not affect HIF-1α at both mRNA and protein level. The values (FOI: Fold of Induction) in the histogram are normalized against GAPDH and are the mean ± SD of three independent experiments. c qPCR demonstrates that in LAMA84 cells curcumin induced a decrease of mRNA IPO7 expression. The values (FOI: Fold of Induction) in the histogram are normalized to GAPDH and are the mean ± SD of three independent experiments. d Representative western blot and corresponding densitogram showing that in LAMA84 cells curcumin inhibited the protein expression of IPO7. e qRT-PCR showing the ability of curcumin to induce in LAMA84 cells a significant increase of miR-22 expression. The values (FOI: Fold of Induction) in the histogram are normalized against RNU6-2 and are the mean ± SD of two independent experiments. In the Western blot assay, actin was used as loading control. Intensities of proteins bands were calculated from the peak area of densitogram by using Image J software. Ctrl: control cells. Statistical significance was calculated vs Ctrl: *p<0.05, **p<0.01.
