# Supplementary material for: SWATH-MS based quantitative proteomics analysis reveals that curcumin alters the metabolic enzyme profile of CML cells by affecting the activity of miR-22/IPO7/HIF-1α axis
Source: J Exp Clin Cancer Res. 2018 Jul 25;37:170. doi: 10.1186/s13046-018-0843-y (PMC6060558; doi:10.1186/s13046-018-0843-y)
Supplement: Supplementary file 10 — Figure S5. Representative western blots and corresponding densitograms showing that in K562 (a) and LAMA84 cells (b) curcumin decreased nuclear levels of HIF-1α. Ponceau S of nuclear extract was used as loading control. Intensities of proteins band (in Ponceau S the band used is indicated with arrow) were calculated from the peak area of densitogram by using Image J software. Ctrl: control cells. (PPTX 809 kb) [file 13046_2018_843_MOESM10_ESM.pptx]

## Slide 1
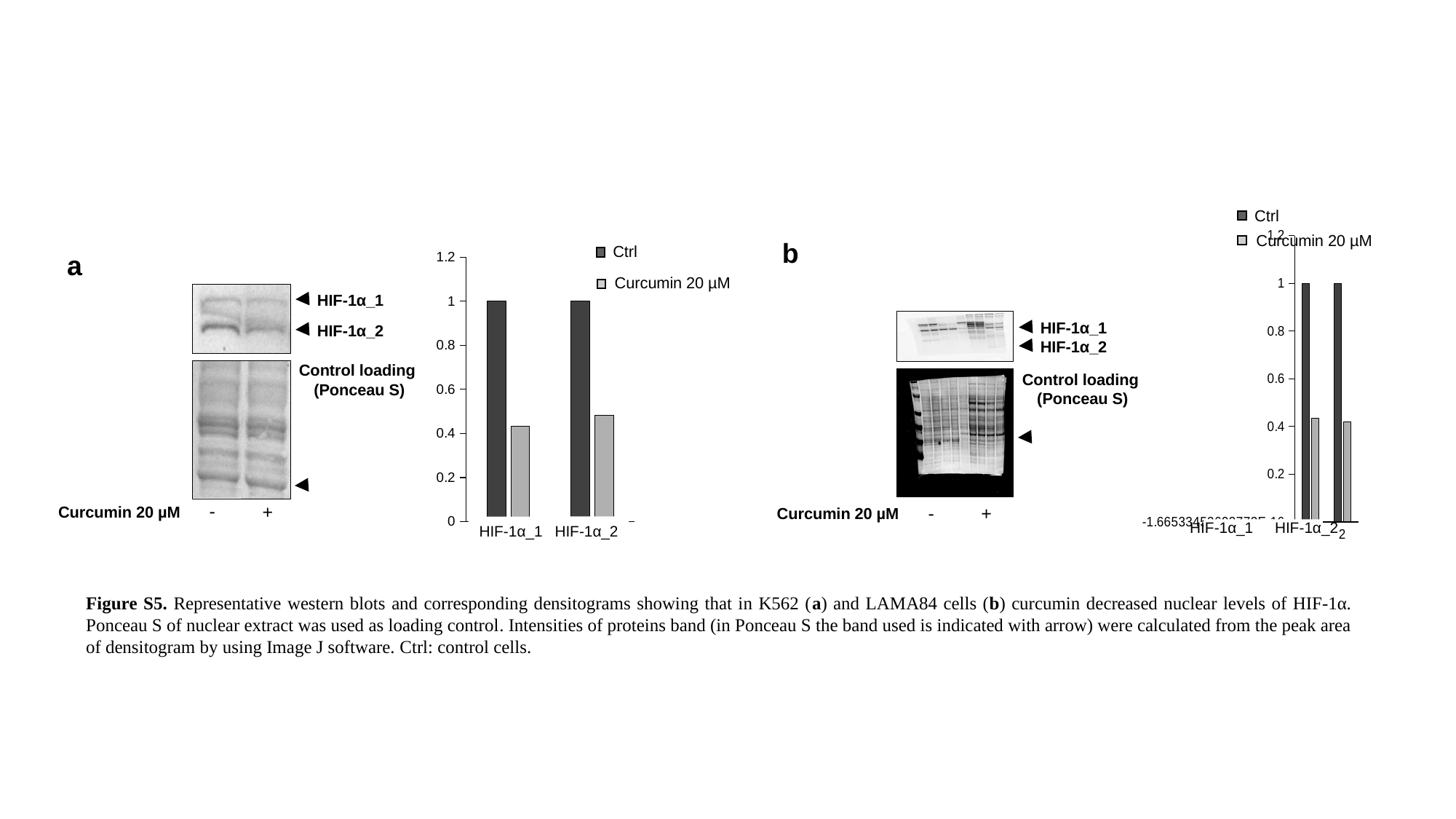

Ctrl
Curcumin 20 µM
### Chart
| Category | | |
|---|---|---|
HIF-1α_1
HIF-1α_2
b
HIF-1α_1
HIF-1α_2
Control loading
(Ponceau S)
-
+
Curcumin 20 µM
Ctrl
Curcumin 20 µM
### Chart
| Category | | |
|---|---|---|HIF-1α_2
HIF-1α_1
a
HIF-1α_1
HIF-1α_2
Control loading
(Ponceau S)
-
+
Curcumin 20 µM
Figure S5. Representative western blots and corresponding densitograms showing that in K562 (a) and LAMA84 cells (b) curcumin decreased nuclear levels of HIF-1α. Ponceau S of nuclear extract was used as loading control. Intensities of proteins band (in Ponceau S the band used is indicated with arrow) were calculated from the peak area of densitogram by using Image J software. Ctrl: control cells.
