# Supplementary material for: SWATH-MS based quantitative proteomics analysis reveals that curcumin alters the metabolic enzyme profile of CML cells by affecting the activity of miR-22/IPO7/HIF-1α axis
Source: J Exp Clin Cancer Res. 2018 Jul 25;37:170. doi: 10.1186/s13046-018-0843-y (PMC6060558; doi:10.1186/s13046-018-0843-y)
Supplement: Supplementary file 11 — Figure S6. IPO7/miRNAs correlation. a Analysis performed by using microRNA target prediction software miRSearch V3.0 showed that IPO7 is a validated target of miR-22 and miR-9. b Analysis of predicted multiple targets performed by MicroRNA Target prediction (miRTar) tool (http://mirtar.mbc.nctu.edu.tw/human/) revealed within the CurcuDown-Regulated dataset the presence of several of miR-22 targets beside IPO7. No target of miR-9 was found. (PPTX 179 kb) [file 13046_2018_843_MOESM11_ESM.pptx]

## Slide 1
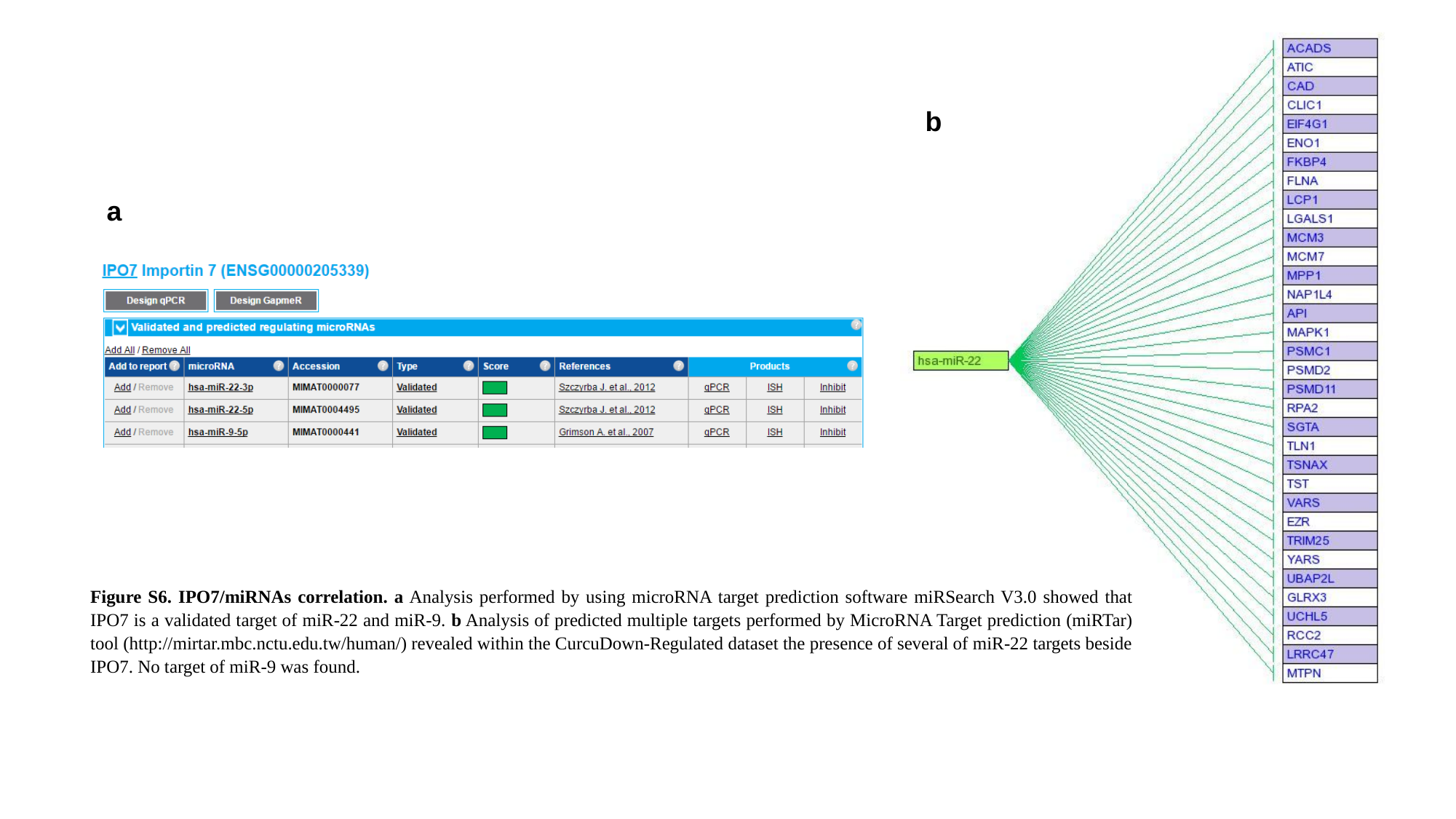

b
a
Figure S6. IPO7/miRNAs correlation. a Analysis performed by using microRNA target prediction software miRSearch V3.0 showed that IPO7 is a validated target of miR-22 and miR-9. b Analysis of predicted multiple targets performed by MicroRNA Target prediction (miRTar) tool (http://mirtar.mbc.nctu.edu.tw/human/) revealed within the CurcuDown-Regulated dataset the presence of several of miR-22 targets beside IPO7. No target of miR-9 was found.
