# Supplementary material for: SWATH-MS based quantitative proteomics analysis reveals that curcumin alters the metabolic enzyme profile of CML cells by affecting the activity of miR-22/IPO7/HIF-1α axis
Source: J Exp Clin Cancer Res. 2018 Jul 25;37:170. doi: 10.1186/s13046-018-0843-y (PMC6060558; doi:10.1186/s13046-018-0843-y)
Supplement: Supplementary file 12 — Figure S7. Anti-proliferative effects of curcumin, imatinib and curcumin+imatinib combination on CML cell viability. Curcumin and imatinib were tested for their anti-proliferative effects on K562 (a) and LAMA84 cells (b). The assays were performed by using curcumin and imatinib singly (using the reported doses) or in combination (20 μM curcumin held constant and imatinib at reported concentrations. In K562 cells combination compound treatments showed significant differences compared to single imatinib treatments for all doses tested (p < 0.001). In LAMA84 cells combination compound treatments showed significant differences compared to single imatinib treatments for lower doses tested (p < 0.001 at 0.1 and 0.2 μM), while no significant differences were observed between combination compound and imatinib at 0.5–5 μM because high cell death occurred. Combination Index (CI) analysis of growth inhibition in K562 (c) and LAMA84 cells (d) after 48 h incubation using curcumin (20 μM) and imatinib (different concentrations). Data from Fig. S6a and S6b were converted to Fraction Affected (FrAf) and plotted against Combination Index (CI). Results were as follows for imatinib concentration: ▲ = 0.1 μM; ♦ = 0.2 μM; ● = 0.5 μM; □ = 1 μM; ○ = 5 μM. Straight line on the graph designates a CI equal to 1. Combination Index interpretation was as follows: CI value of 1 indicates additivity; CI < 1 indicates synergism; and CI > 1 indicates antagonism. (PPTX 50 kb) [file 13046_2018_843_MOESM12_ESM.pptx]

## Slide 1
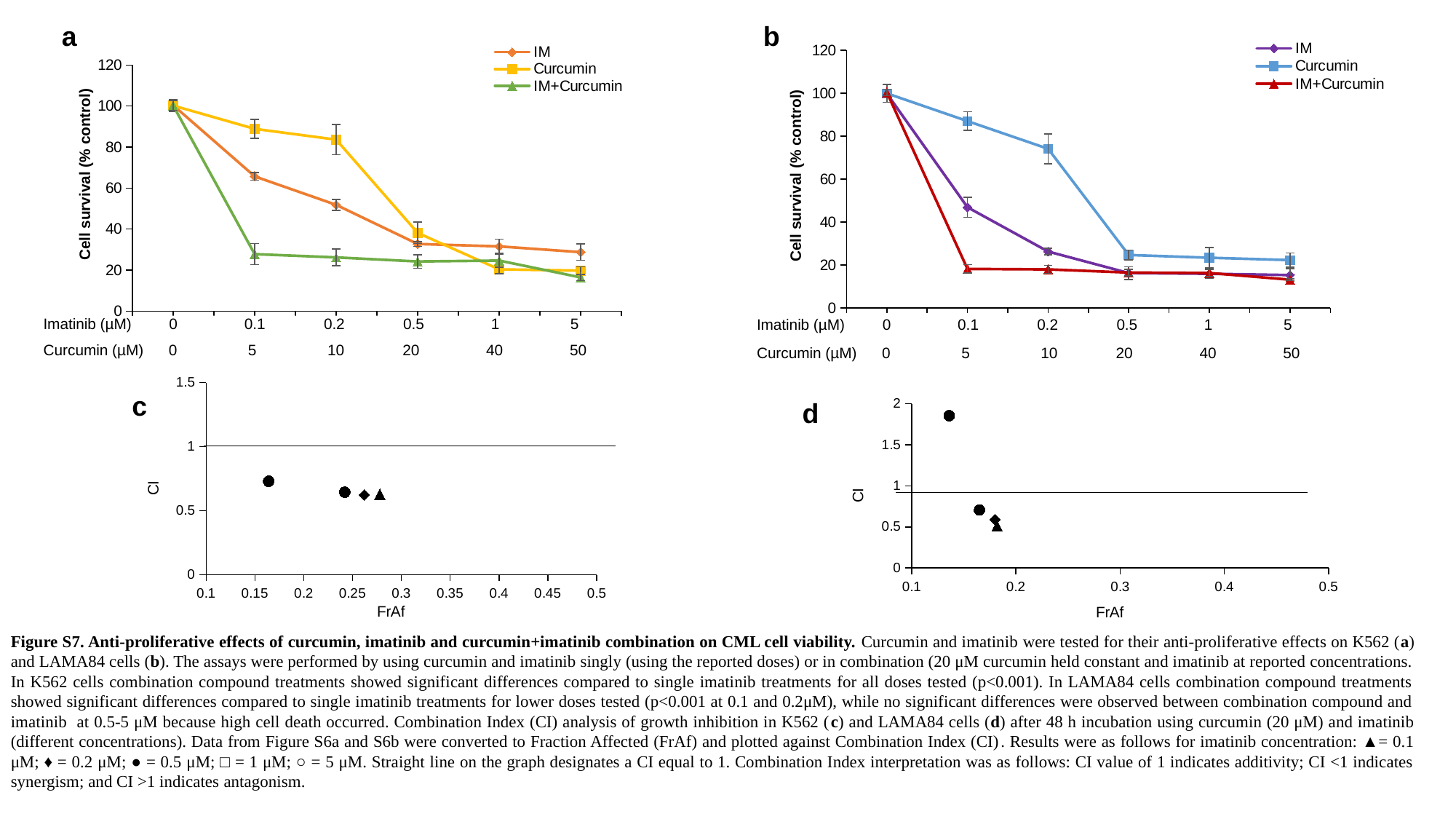

a
### Chart
| Category | IM | Curcumin | IM+Curcumin |
|---|---|---|---|Imatinib (µM) 0 0.1 0.2 0.5 1 5
Curcumin (µM) 0 5 10 20 40 50
b
### Chart
| Category | IM | Curcumin | IM+Curcumin |
|---|---|---|---|Imatinib (µM) 0 0.1 0.2 0.5 1 5
Curcumin (µM) 0 5 10 20 40 50
Cell survival (% control)
Cell survival (% control)
### Chart
| Category | CI |
|---|---|c
d
### Chart
| Category | CI |
|---|---|Figure S7. Anti-proliferative effects of curcumin, imatinib and curcumin+imatinib combination on CML cell viability. Curcumin and imatinib were tested for their anti-proliferative effects on K562 (a) and LAMA84 cells (b). The assays were performed by using curcumin and imatinib singly (using the reported doses) or in combination (20 μM curcumin held constant and imatinib at reported concentrations. In K562 cells combination compound treatments showed significant differences compared to single imatinib treatments for all doses tested (p<0.001). In LAMA84 cells combination compound treatments showed significant differences compared to single imatinib treatments for lower doses tested (p<0.001 at 0.1 and 0.2μM), while no significant differences were observed between combination compound and imatinib at 0.5-5 μM because high cell death occurred. Combination Index (CI) analysis of growth inhibition in K562 (c) and LAMA84 cells (d) after 48 h incubation using curcumin (20 μM) and imatinib (different concentrations). Data from Figure S6a and S6b were converted to Fraction Affected (FrAf) and plotted against Combination Index (CI). Results were as follows for imatinib concentration: ▲= 0.1 μM; ♦ = 0.2 μM; ● = 0.5 μM; □ = 1 μM; ○ = 5 μM. Straight line on the graph designates a CI equal to 1. Combination Index interpretation was as follows: CI value of 1 indicates additivity; CI <1 indicates synergism; and CI >1 indicates antagonism.
